# Effects of combining constraint-induced movement therapy and action-observation training on upper limb kinematics in children with unilateral cerebral palsy: a randomized controlled trial

**DOI:** 10.1038/s41598-020-67427-2

**Published:** 2020-06-26

**Authors:** Cristina Simon-Martinez, Lisa Mailleux, Ellen Jaspers, Els Ortibus, Kaat Desloovere, Katrijn Klingels, Hilde Feys

**Affiliations:** 1 Department of Rehabilitation Sciences, KU Leuven, 3000 Leuven, Belgium; 2 Information Systems Institute, University of Applied Sciences Western Switzerland (HES-SO Valais), Sierre, Switzerland; 3 Neural Control of Movement Lab, ETH Zurich, Zurich, Switzerland; 4 Department of Development and Regeneration, KU Leuven, Leuven, Belgium; 5 Clinical Motion Analysis Laboratory, University Hospitals Leuven, Leuven, Belgium; 6 Rehabilitation Research Centre, Faculty of Rehabilitation Sciences, Hasselt University, Diepenbeek, Belgium

**Keywords:** Health care, Paediatrics, Therapeutics, Randomized controlled trials, Outcomes research, Paediatric research

## Abstract

Modified constraint-induced movement therapy (mCIMT) improves upper limb (UL) motor execution in unilateral cerebral palsy (uCP). As these children also show motor planning deficits, action-observation training (AOT) might be of additional value. Here, we investigated the combined value of AOT to mCIMT on UL kinematics in children with uCP in a randomized controlled trial. Thirty-six children with uCP completed an UL kinematic and clinical evaluation after participating in a 9-day mCIMT camp wearing a splint for 6 h/day. The experimental group (mCIMT + AOT, n = 20) received 15 h of AOT, i.e. video-observation and execution of unimanual tasks. The control group (mCIMT + placebo, n = 16) watched biological-motion free videos and executed the same tasks. We examined changes in motor control (movement duration, peak velocity, time-to-peak velocity, and trajectory straightness) and kinematic movement patterns (using Statistical Parametric Mapping) during the execution of three unimanual, relevant tasks before the intervention, after and at 6 months follow-up. Adding AOT to mCIMT mainly affected movement duration during reaching, whereas little benefit is seen on UL movement patterns. mCIMT, with or without AOT, improved peak velocity and trajectory straightness, and proximal movement patterns. Clinical and kinematic improvements are poorly related. Although there seem to be limited benefits of AOT to CIMT on UL kinematics, our results support the inclusion of kinematics to capture changes in motor control and movement patterns of the proximal joints.

## Introduction

The upper limb (UL) motor and sensory deficits in children with unilateral Cerebral Palsy (uCP) hinder their performance of daily life activities^1^. Effective rehabilitation approaches include bimanual training^2^, modified constraint-induced movement therapy^3^, or goal-directed training^4^. One of the most popular treatment modalities amongst clinicians and researchers is constraint-induced movement therapy (CIMT) and its modified versions (mCIMT)^5^, due to its proven high effectiveness and its ability to improve both unimanual and bimanual function^6–9^. mCIMT consists of constraining the less impaired hand while targeting intensive unimanual task-related practice with the more impaired UL^10^.

Apart from motor execution problems, children with uCP may also have difficulties in motor representation and motor planning^11,12^. These deficits can be targeted with Action-Observation Training (AOT), a novel treatment approach aimed at activating the mirror neuron system. Mirror neurons are a particular class of visuomotor neurons that discharge both when we execute a particular motor action, and when we observe another individual executing the same action^13^. It seems that through observation, we may learn how to imitate the movements required to perform a specific action^14,15^, which can be translated into skill learning. A few studies have already shown the efficacy of AOT in improving UL sensorimotor deficits in children with uCP^16–20^. However, the added value of AOT to a well-established rehabilitation approach, such as mCIMT, has not yet been investigated.

Changes in UL sensorimotor function following mCIMT or AOT have been typically evaluated with clinical scales that rely on an ordinal-based (subjective) scoring system. Three-dimensional motion analysis (3DMA) provides a quantitative measurement of UL kinematics (spatiotemporal characteristics and joint angles, indicative of motor control and movement patterns) of proximal and distal joints^21–23^, highlighting its added value compared to clinical scales^24^. 3DMA has shown that children with uCP perform unimanual tasks with longer duration, lower and earlier peak velocity, and with a less straight trajectory^21^ and with aberrant movement patterns at all levels of the UL chain^21,25^, compared to typically developing children. Previous studies have shown the utility and sensitivity of a 3DMA to identify improvements at joint level after UL surgery and botulinum toxin injections^26,27^. These improvements were most obvious in the proximal joints (shoulder, scapula or trunk) for which clinical scales are less sensitive^26^. The analysis of waveforms derived from 3DMA data using Statistical Parametric Mapping (SPM1d) has allowed us to accurately map UL deficits in children with uCP^25^. Treatment-induced changes of UL movement pathology require a comprehensive analysis over the entire waveform of the joint angle, and the use of SPM1d on 3DMA data will potentially enable us to capture changes in movement patterns after an intensive training.

Although both mCIMT and AOT have individually been shown to be efficient in improving UL function in children with uCP, it remains unknown whether their combination would be more effective than mCIMT alone. Furthermore, the comprehensive evaluation of UL movement patterns obtained with 3DMA could be highly beneficial to capture training-induced changes, especially at the joint level. Therefore, the aim of this study was to examine the added value of AOT to mCIMT on improving UL kinematics (motor control and movement patterns), as measured with UL-3DMA. Additionally, we aimed to explore the relation between the clinical and the kinematic improvements, to further validate the UL-3DMA assessment. Considering the exploratory nature of this study, we hypothesized that the addition of AOT will result in (i) better motor control in terms of shorter movement duration, higher and later peak velocity, and a straighter trajectory path; (ii) improved joint angles (related to typically developing children^25^) especially distally, as the AOT focuses on hand movements.

## Materials and methods

This RCT protocol has been previously described in detail^28^, and will be briefly summarized here. The study was conducted at KU Leuven and was approved by the Ethics Committee Research UZ / KU Leuven (S56513), in agreement with the Declaration of Helsinki. All participants assented to participate, and their parents or caregivers signed the informed consent. The study is registered at www.clinicaltrials.gov (identifier NCT03256357, registered on 22nd August 2017). There have been no changes in the trial protocol after the trial commencement.

### Study population and randomization

Children with uCP aged 6–12 years were recruited via the CP reference centre of the University Hospitals Leuven, by CSM and LM. Children were included if they had a score between 4 (i.e. poor active assist) and 8 (i.e. spontaneous use) according to the House Functional Classification (HFC) scale^29^, and sufficient cooperation to complete the test procedure. Exclusion criteria were UL surgery 2 years prior to testing or UL botulinum toxin A injections 6 months prior to testing.

Sample size was estimated based on the primary endpoint, which is defined as the immediate effect of the intervention on the primary outcome measure of the larger study, i.e. bimanual performance measured with the Assisting Hand Assessment^28^. With an estimated effect size of 0.9, an alpha-level of 0.05, and a statistical power of 0.80, a sample size of 21 children is needed in each group to detect a difference equal to or larger than the smallest detectable difference of 5 AHA units between groups^28,30^.

Between 2014 and 2017, we organized seven summer camps (July and August) in which the children were prospectively enrolled. The overview and more detailed information about the camps (location and number of participants) as well as demographic and clinical characteristics of the participants in each camp can be found Supplementary Materials (Table S1) of Simon-Martinez et al.^31^. Participants were first stratified according to the HFC scale (4–5 vs. 6–7), age (6-9y vs. 10–12 years), and the type of corticospinal tract wiring pattern (contralateral, bilateral and ipsilateral as measured with transcranial magnetic stimulation). Next, participants were allocated to the experimental (mCIMT + AOT) or the control (mCIMT + placebo) group, by using a permuted block design of two. The randomization was conducted by an independent researcher (HF). The trial was ended when the data collection was completed.

### Intervention

The intervention consisted of mCIMT with AOT or placebo and it was delivered in a day camp model for 9 out of 11 consecutive days (6 h/day, total of 54 h of therapy), with no therapy during the weekend. One or two camps per year were organized in different locations of Flanders (Belgium), in special education schools or at the sports centre of KU Leuven^31^. To guarantee individual guidance, the therapist-child ratio was 1:1. A team of experienced paediatric physiotherapists led the camps, assisted by paediatric physiotherapy master students. During the camp hours, all children wore a tailor-made hand splint on the less impaired hand while using only the more affected hand during individual therapy or group activities, and during the AOT or placebo condition. The intervention was ramified in three blocks maintaining the principle of unimanual use: individual therapy, group activities and the AOT/placebo sessions.

The *individual therapy* (9 h) was based on motor learning principles of shaping and repetitive practice focussing on: (1) active wrist and elbow extension, (2) forearm supination, (3) grip strength, and (4) fine motor tasks. These goals were embedded in functional tasks, which were tailored to meet the child’s therapeutic needs and adapted according to the child’s progress. To do so, we used materials like modelling compound, board games, cards, magnets, glass bead to do bracelets, among others. The *group activities* (30 h) consisted of painting and crafting (making a grass man, making musical instruments with cardboard), cooking (making juice cocktails, fruit skewers, chocolate fondue), and outdoor games (gymkhana for children including e.g. building towers, making puzzles and water games including filling a bucket with a sponge/bottle, filling and throwing water balloons), specifically selected to stimulate the intensive use of the more-impaired hand. The intervention during individual and group activities was standardized across participants but individualized to each child’s needs. Children assigned to the mCIMT + AOT group received 15 *AOT sessions* (each lasting 1 h), during which they watched video sequences showing unimanual goal-directed, functional actions from the first-person perspective (i.e. as if they were performing the action). All actions included the grasp and/or release of an object, which could be done in different orientations. To maximize the variety of the actions we included different objects (e.g. coins, bottles, stamps, modelling compound) to be grasped with different grasp types (whole hand, pinch, tripod grasp) and in different orientations of the wrist. The videos were recorded with one adult hand without disability. Afterwards, they were mirrored so that the action was exactly the same for both left- and right-side affected children and looped for 3 min. The videos were delivered through a laptop and a 22 inches screen. All actions were adapted to the UL functional level of the child (see Additional files 1 and 2 of^28^). The action was observed for 3 min and afterwards executed for 3 min, which was repeated twice per action, totalling six actions per session. To assure that the children were paying attention to the videos, a yes/no question related to each video activity was asked after the second execution of each activity (e.g. is the box taken from the top? Did you see the palm of the hand?). At the end of the intervention, the number of correct answers were summed up, ranging from 0 (all answers incorrect) to 45 (all answers correct). The children in the mCIMT + placebo group played video games without biological motion (15 h). Afterwards, the therapist explained the same action that was observed by the mCIMT + AOT group and the child executed it for 3 min.

Although treatment fidelity was not measured, the one-to-one therapist-child ratio assured an individualized guidance and motivation to keep the children engaged in the therapy during the camp. Additionally, the use of a rehabilitation robot (Zora robot, https://zorarobotics.be/index.php/en/zorabot-zora) also helped into the daily engagement of the children in the training, by welcoming the children to the camp activities and by encouraging them to do their best on the next day.

### Evaluation

The evaluation consisted in a comprehensive assessment of UL function including clinical and kinematic measures. This study reports additional, secondary outcomes of a larger RCT reported elsewhere^28,31^. The main outcome of this study was UL kinematics, which consisted in an UL-3DMA evaluation, based on a protocol specially developed for children with uCP^23,24^. The protocol has shown to be reliable and valid, and full protocol details can be found elsewhere^23,28^. Seventeen reflective markers were mounted on the trunk, acromion, upper arm, forearm and hand to record UL motion with 12–15 Vicon infrared cameras (Oxford Metrics, Oxford, UK) sampling at 100 Hz. Children sat in a custom-made chair that allowed standard sitting with foot and back support. We identified the anatomical landmarks of interest with static calibration trials following the International Society of Biomechanics guidelines^32^. Our original protocol involved children completed eight unimanual tasks^28^. Our subsequent research identified that three of these eight unimanual tasks are adequate to depict pathological movement patterns compared to typically developing peers, which would be affected by increased tone and decreased strength in children with uCP^25^. Therefore, the movement protocol of this RCT included those three tasks: reaching upward (RU), reach-to-grasp a vertically oriented cylinder (RGV), and hand-to-shoulder (HTS). Detailed description on the performance of these tasks are freely available on GitHub (https://github.com/u0078867/ulema-ul-analyzer/blob/master/AppendicesI-II.pdf) and marker location at start and end position can be found in Fig. S1 of Supplementary Materials. Children were instructed to perform each task four times at self-selected speed, in two different trials, resulting in 8 movement repetitions per task. Children were evaluated right before (weekend before the start of the camp, T1) and after (weekend after the start of the camp, T2) the intervention, as well as at 6 months follow-up (T3), as this period has been shown to still capture long-term effects of the intervention^31^. LM and CSM conducted the UL-3DMA evaluations.

To report the clinical characteristics of the participants, we additionally included an evaluation of muscle tone, muscle weakness and grip force, aiming to explore whether the improvements in the clinical evaluation are correlated with improvements in kinematics. The effects of the intervention on these clinical measures has been published elsewhere^31^. We used the Modified Ashworth Scale (MAS^33^) and the 8-point ordinal scale of the Medical Research Council^34^ to assess UL muscle tone and muscle weakness, respectively. Children performed three maximum contractions with the Jamar dynamometer (Sammons Preston, Rolyan, Bolingbrook, IL, USA) to evaluate grip strength and the mean was taken for further analyses. More details about this evaluation can be found elsewhere (Supplementary Materials and methods section of Simon-Martinez et al.^31^). The clinical evaluation was performed by an experienced physiotherapist blinded to group-allocation.

### Data processing

Offline data processing was performed using Vicon Nexus software (version 1.8.5, Oxford Metrics, Oxford, UK) and included a Woltring filtering routine with a predicted mean squared error of 10 mm^2^^35^, gap filling, and selection of the movement cycles (start and endpoints, see Supplementary Materials Fig. S1 for marker location at these positions for each task). Only the middle two repetitions of each trial were time-normalized (0–100%) and used for the kinematic analysis. The open source software U.L.E.M.A v1.1.9^23,36,37^ was used to retain the three cycles with the lowest root mean square error for the computation of spatiotemporal parameters and joint angles. To investigate changes in *motor control*, we extracted spatiotemporal parameters from the central hand marker and included movement duration (seconds), peak velocity (m/s), time-to-peak velocity (% of the cycle), and trajectory straightness (unit less). A higher peak velocity indicates greater force or impulse during the movement^38,39^. An earlier time-to-peak velocity points to the used movement strategy, dividing the movement in ‘before’ (time spent during the first visually triggered outward movement) and ‘after’ the peak velocity (the second half of the movements requires more precision to be successful)^40^. Lastly, trajectory straightness indicates movement efficiency (i.e. movement smoothness) and is calculated as the ratio of the actual length of the travelled hand marker path and the direct linear distance between start and endpoint. To investigate changes in *movement patterns*, joint angles were calculated for the trunk (axial rotation, lateral flexion, and flexion–extension), scapula (medial–lateral rotation, tilting, and pro-retraction), shoulder (internal–external rotation, elevation plane, and elevation), elbow (flexion–extension and pro-supination), and wrist (flexion–extension).

### Statistical analyses

Spatiotemporal parameters were checked with the Shapiro–Wilk test and their histograms were checked for symmetry. The data was shown to be normally distributed and therefore descriptive statistics are reported as mean and standard errors of the mean and parametric statistics were used. A repeated-measures Analysis of Variance (rmANOVA) was conducted to evaluate changes over time (T1, T2, and T3) and between groups (mCIMT + AOT vs. mCIMT + placebo). The time*group interaction was included in the model to explore potential different time trends between groups. In case of a significant interaction, time trends were investigated separately per group. Otherwise, time trends were further investigated in both groups combined. No imputation was made for missing participant data and participants were excluded from analysis for the variable with missing participant data at that time point. The alpha-level for interaction and main effects for the spatiotemporal parameters was set at 0.05, with a post-hoc Tukey HSD correction. Effect sizes are reported for the comparison of the spatiotemporal parameters as partial η^2^ in the rmANOVA models (small 0.01; medium 0.06; large 0.14)^41,42^ and as Cohen’s d effect sizes in the pair-wise comparisons (small, 0.2–0.5; medium, 0.5–0.8; large > 0.8)^42^. The statistical analysis of the spatiotemporal data was performed in SPSS (version 25.0, SPSS Inc, Chicago, IL, USA).

The waveform analysis of joint angles was conducted using the Statistical Parametric Mapping (SPM) for 1 dimensional data toolbox (SPM1d, version 0.4 for Matlab, available for download at https://www.spm1d.org/Downloads.html)^43^. SPM1d allows for hypothesis testing over the entire spectrum by considering the interdependency of the data points using random field theory and thus reduces the risk of type I errors. For every joint angle, the waveforms are compared using the conventional univariate statistic of an rmANOVA, outputting a statistical curve (F-curve). Next, random field theory is applied to estimate the critical threshold above which only 5% of equally smoothed random data is expected to cross (α < 0.05). When clusters (i.e. differences between groups or over time) are identified, the location, extent, and a single p-value is extracted. Clusters smaller than 5% of the movement cycle are not reported due to little clinical relevance. For every joint angle, we first tested the time*group interaction to evaluate different time trends between groups. If the interaction was significant, time trends were investigated in each group. Otherwise, time trends were investigated for both groups combined. For the SPM1d post-hoc comparisons, alpha-level for interaction and main effects was set at 0.05, and Bonferroni-corrected for post-hoc analysis (division by the number of comparisons (i.e. 0.05/3)).

Finally, to evaluate whether there was an association between the improvements in motor control parameters and clinical improvement, we ran correlation analyses between the change of these variables using Spearman correlations, due to the ordinal characteristics of the clinical measures. We ran the correlation analyses for change between T1-T2 and between T1-T3. Correlation analyses between clinical and kinematic measures were interpreted as little or no correlation (< 0.30), low (0.30–0.50), moderate (0.50–0.70), high (0.70–0.90), and very high correlation (> 0.90)^44^. Alpha-level for these correlation analyses was set at 0.01 to correct for the multiple number of correlations.

## Results

### Participants

**Figure 1:**
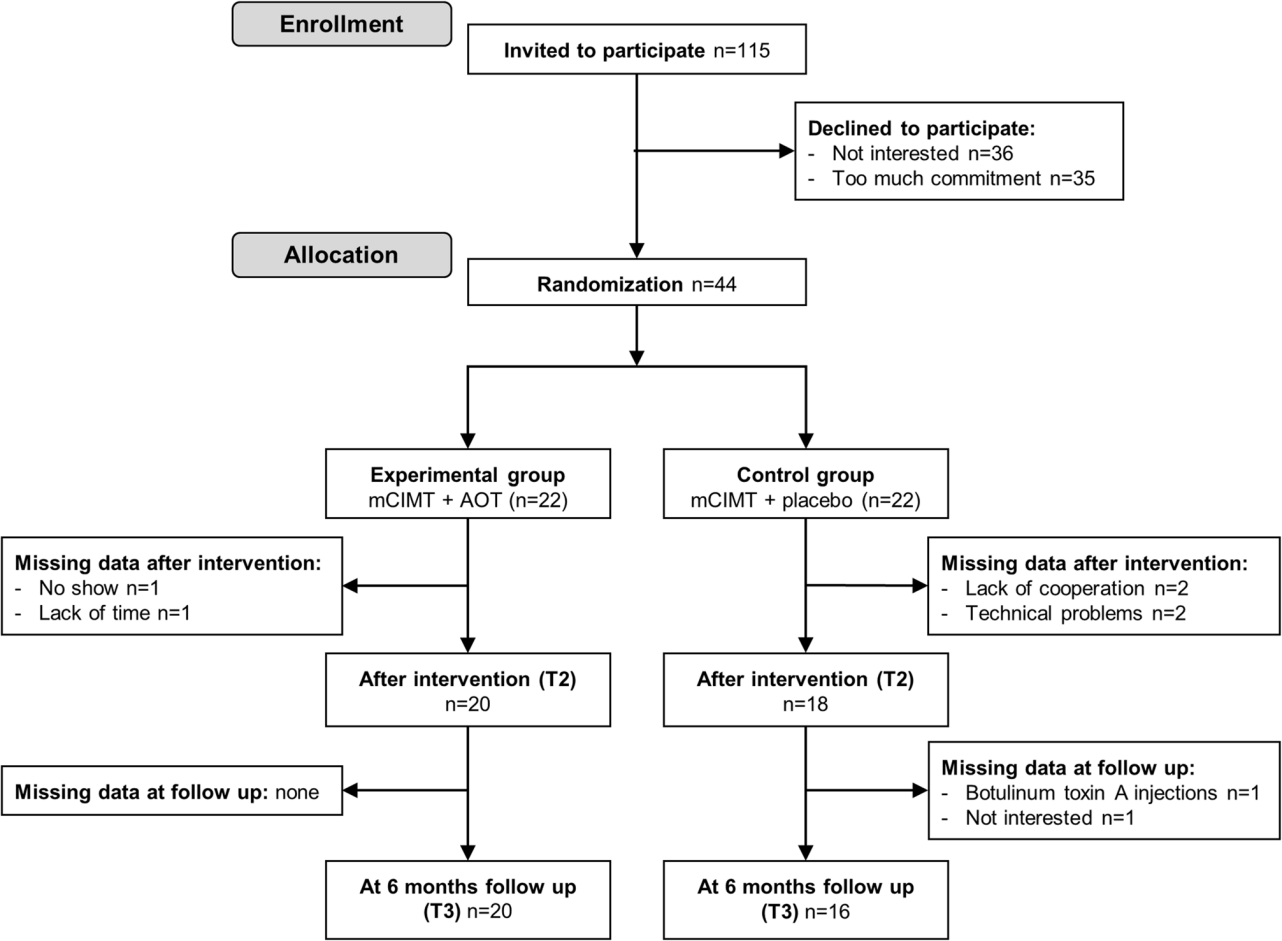
CONSORT flowchart with number of participants and reasons for missing data in each group, at each time-point.

Forty-four children with uCP participated in this study (mean age (SD) 9y6m (1y10m); 27 boys; 9 MACS I, 15 MACS II, 20 MACS III), and were randomized into the mCIMT + AOT (n = 22) and the mCIMT + placebo (n = 22). Baseline characteristics of the randomized participants are reported in Table 1. In the mCIMT + AOT group, data of two children were missing immediately after the intervention. In the mCIMT + placebo group, data of four children were missing immediately after the intervention and data of two were missing at follow-up, resulting in a total of 36 children of whom n = 20 received mCIMT + AOT and n = 16 mCIMT + placebo (Fig. 1). Baseline characteristics of the participants included in the final analysis (excluding participants with missing data) are reported in Supplementary Materials (Table S1). All participants completed the intervention and children who received AOT showed a good compliance to the video observation, based on the number of correct answers to the video-related questions (median = 42/45 correctly answered, interquartile range = 5, range 30–45). In the camps, children were compliant to all the training period by attending the camp every day. There were few cases when a child might have been frustrated while performing the unimanual activities and the cooperation decreased. However, this was quickly solved by decreasing the task difficulty or by talking to the child to find a way to keep his/her compliance in the training.

**Table 1:** Demographic characteristics of the randomized participants per group and statistical comparison of the demographic characteristics.

	CIMT + AOT (n = 22)	CIMT + placebo (n = 22)	p-value
Age			
Mean (SD)	9y6m (1y11m)	9y6m (1y10m)	0.89^a^
Sex, n (%)			
Boys	15 (68)	12 (55)	0.35^b^
Girls	7 (32)	10 (45)	
More affected side, n (%)			
Left	9 (41)	14 (64)	0.13^b^
Right	13 (59)	8 (36)	
MACS, n (%)			
I	6 (27)	3 (14)	0.39^b^
II	8 (36.5)	7 (32)	
III	8 (36.5)	12 (55)	
HFC system, n (%)			
Levels 4–5	16 (73)	18 (82)	0.47^b^
Level 6–8	6 (27)	4 (18)	

*MACS* manual ability classification system, *HFC* house functional classification.

^a^Independent samples t-test.

^b^Pearson Chi-Square test.

At T1, the children included in the analyses of the current manuscript also showed deficits in muscle tone (mCIMT + AOT group, mean 5.26, SD 1.29; mCIMT + placebo group, mean 5.13, SD 2.07), muscle weakness (mCIMT + AOT group, mean 10.15, SD 1.18; mCIMT + placebo group, mean 10.16, SD 0.63) and grip force (mCIMT + AOT group, mean 4.95, SD 3.13; mCIMT + placebo group, mean 5.30, SD 4.51), typical from children with uCP. A more detailed report of the clinical evaluation can be found elsewhere^31^. These deficits were not different between groups at T1 (all p > 0.05).

### Treatment efficacy

The effects of this RCT will be first reported for between-group differences to evaluate the added value of AOT to mCIMT. Next, the within-group effects over time will be described for the total group, to evaluate the effects of mCIMT with or without AOT. There were no harms or unintended effects in either group.

### Effects of the addition of AOT to mCIMT on kinematic measures

**Figure 2:**
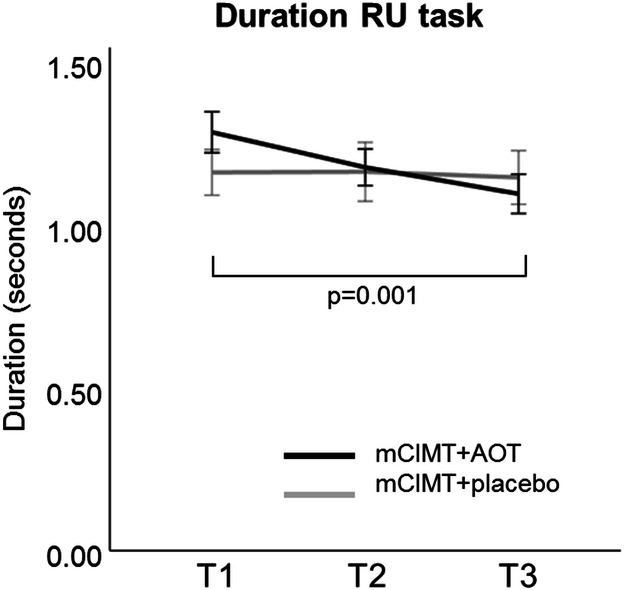
Movement duration during task reach upwards (RU). The mCIMT + AOT group improved more than the control group. Differences were significant at 6 months follow-up.

Table 2 summarizes the results for spatiotemporal parameters for each group (mCIMT + AOT vs. mCIMT + placebo) at every time point (T1, T2, and T3). We found no between-group differences at baseline (T1) for any spatiotemporal parameter (all p > 0.05). We found a significant time*group interaction for movement duration during RU (F = 3.37, p = 0.04, partial η^2^ = 0.17) in favour of the mCIMT + AOT group (F = 9.83, p = 0.001). In this group, post-hoc analysis showed significant improvement at follow-up (T1-T3, p = 0.001, d = 0.64) (Fig. 2). The mCIMT + placebo group showed no significant improvements in movement duration (p > 0.05). No significant interactions were found for the other spatiotemporal parameters or the other tasks (p > 0.05).

**Table 2:** Estimated marginal means (standard error) of spatiotemporal parameters (indicative of motor control) at each time-point, and statistical comparison [F (p-values; effect size)].

	T1 (pre)	T2 (post)	T3 (6 m follow up)	Time*Group (F (p; partial η^2^))	Time (groups combined) (F (p; partial η^2^))
**RU—duration (s)**					
mCIMT + placebo	1.17 (0.07)	1.17 (0.08)	1.15 (0.08)	**3.37 (0.04; 0.17)**	
mCIMT + AOT	1.29 (0.06)	1.19 (0.07)	1.10 (0.07)		
**RGV—duration (s)**					
mCIMT + placebo	1.87 (0.13)	1.70 (0.13)	1.71 (0.15)	0.12 (0.89; 0.01)	3.03 (0.06; 0.15)
mCIMT + AOT	1.82 (0.12)	1.67 (0.12)	1.62 (0.13)		
**HTS—duration (s)**					
mCIMT + placebo	1.37 (0.10)	1.37 (0.10)	1.40 (0.09)	0.65 (0.53; 0.04)	0.20 (0.82; 0.01)
mCIMT + AOT	1.28 (0.09)	1.29 (0.09)	1.20 (0.08)		
**RU—trajectory**					
mCIMT + placebo	1.40 (0.04)	1.42 (0.04)	1.39 (0.04)	0.75 (0.48; 0.04)	2.92 (0.07; 0.15)
mCIMT + AOT	1.48 (0.03)	1.52 (0.04)	1.44 (0.03)		
**RGV—trajectory**					
mCIMT + placebo	1.42 (0.04)	1.39 (0.04)	1.39 (0.04)	0.79 (0.47; 0.05)	2.92 (0.07; 0.15)
mCIMT + AOT	1.46 (0.04)	1.45 (0.04)	1.40 (0.04)		
**HTS—trajectory**					
mCIMT + placebo	1.58 (0.04)	1.56 (0.05)	1.50 (0.04)	0.21 (0.81; 0.01)	**3.52 (0.04; 0.17)**
mCIMT + AOT	1.53 (0.04)	1.53 (0.05)	1.47 (0.04)		
**RU—peak velocity (m/s)**					
mCIMT + placebo	1.38 (0.07)	1.43 (0.07)	1.50 (0.07)	0.20 (0.82; 0.01)	**5.13 (0.01; 0.23)**^bc^
mCIMT + AOT	1.35 (0.06)	1.37 (0.07)	1.48 (0.06)		
**RGV—peak velocity (m/s)**					
mCIMT + placebo	1.03 90.05)	1.08 (0.06)	1.10 (0.05)	0.14 (0.87; 0.01)	**5.67 (0.008; 0.25)**^ab^
mCIMT + AOT	0.97 (0.05)	1.04 (0.05)	1.06 (0.05)		
**HTS—peak velocity (m/s)**					
mCIMT + placebo	1.04 (0.05)	1.07 (0.05)	1.03 (0.05)	0.73 (0.49; 0.04)	0.21 (0.81; 0.01)
mCIMT + AOT	1.04 (0.05)	0.99 (0.05)	1.01 (0.04)		
**RU—time to peak velocity (% cycle)**					
mCIMT + placebo	35.49 (1.59)	32.73 (1.26)	35.44 (2.01)	0.57 (0.57; 0.03)	2.11 (0.14; 0.11)
mCIMT + AOT	33.63 (1.42)	31.66 (1.13)	31.90 (1.80)		
**RGV—time to peak velocity (% cycle)**					
mCIMT + placebo	23.90 (1.50)	23.07 (1.32)	22.69 (1.71)	0.59 (0.56; 0.04)	0.36 (0.70; 0.02)
mCIMT + AOT	24.18 (1.34)	23.38 (1.18)	25.00 (1.53)		
**HTS—time to peak velocity (% cycle)**					
mCIMT + placebo	35.23 (2.01)	31.88 (2.51)	32.37 (2.39)	1.54 (0.23; 0.09)	0.05 (0.95; 0.003)
mCIMT + AOT	32.25 (1.85)	35.06 (2.25)	35.36 (2.14)		

*RU* reach upwards, *RGV* reach-to-grasp a vertically oriented cylinder, *HTS* hand to shoulder, *mCIMT* modified constraint-induced movement therapy, *AOT* action-observation training, *Vmax* maximum velocity.

^a^Significant for T1 vs. T2.

^b^Significant for T1 vs. T3.

^c^Significant for T2 vs. T3.

A summary of the findings based on the SPM1d analyses of the kinematic waveforms are reported in Table 3. We found time*group interactions for shoulder (all tasks), scapula (HTS), and wrist (HTS) kinematics, whilst no time*group interactions were found for the movement patterns of the trunk and elbow (p > 0.05).

**Table 3:** Statistical parametric mapping results of the effect of the intervention (Time*Group in bold; Time with both groups combined without shading) over time, for each joint angle, for the three tasks.

	Reaching upward (RU)		Reach-to-grasp a vertically oriented cylinder (RGV)		Hand-to-shoulder (HTS)	
	Location (extent)	p-value	Location (extent)	p-value	Location (extent)	p-value
**Trunk**						
Flexion–extension						
Lateral flexion	67–100% (33%)	0.03				
Axial rotation					58–100% (42%)	0.02
**Scapula**						
Tilting					**18–100% (82%)**	**0.007**^**b**^
Pro-retraction						
Med-Lat rotation	15–100% (85%)	0.002	14–100% (86%)	0.002		
**Shoulder**						
Elevation			**0–22% (22%)**	**0.03**^**a**^	**0–33% (33%)**	**0.02**^**c**^
Elevation plane						
Int-Ext rotation	**47–100% (53%)**	**0.02**^**a**^	**8–31% (23%)**	**0.04**^**a**^	**0–32% (32%)**	**0.03**^**a**^
**Elbow**						
Flexion–extension						
Pro-supination						
**Wrist**						
Flexion–extension					**7–17% (10%)**	**0.047**^**c**^

Significant results of the time*group interaction are presented in bold shading. Main effects when the interaction was not significant (for both groups combined) are shown without shading.

^a^Main effects within each group not significant.

^b^Main effects significant in the mCIMT + AOT group.

^c^Main effects significant in the mCIMT + placebo group.

**Figure 3:**
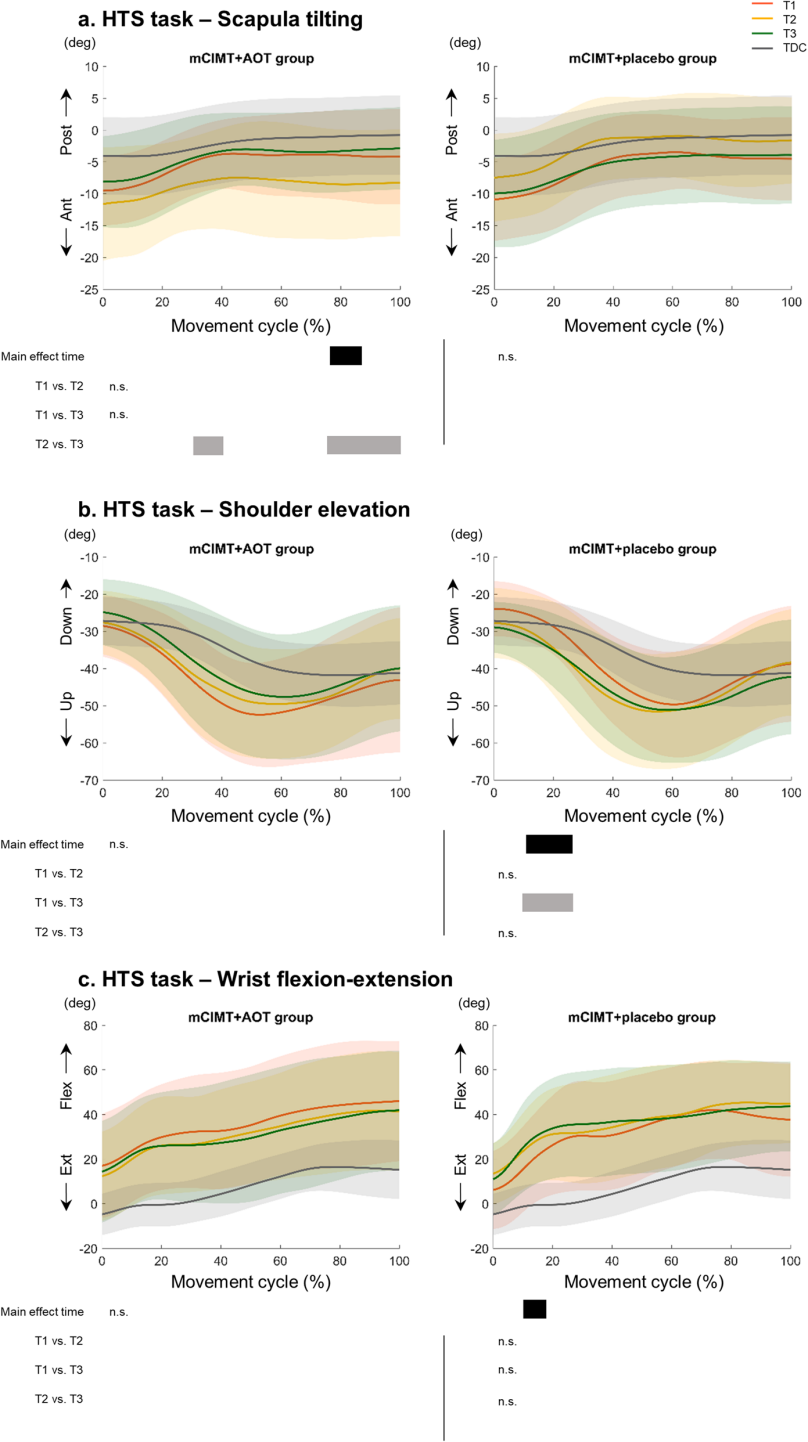
Time*group interactions for (**A**) scapula tilting, (**B**) shoulder elevation, and (**C**) wrist flexion–extension during the performance of the hand-to-shoulder task. Mean and standard deviation of the mCIMT + AOT group (left panel) and mCIMT + placebo group (right panel) is shown. Each subpanel displays where over the waveform the main effects (black bars) and the post-hoc analyses (grey) in each group were depicted. *n.s.* not significant.

For the shoulder, further analysis showed no significant differences in any of the intervention groups for *shoulder rotation* (all tasks) and *shoulder elevation* (RGV). The analysis for *shoulder elevation* also showed a time*group interaction (HTS: p = 0.02, 0–33% of the movement cycle), whereby the mCIMT + placebo group performed the task with less shoulder elevation over time (p = 0.04, 11–24% of the movement cycle), whilst the mCIMT + AOT group did not show changes over time (p > 0.05, Fig. 3b). Post-hoc analyses in the mCIMT + placebo group indicated that the improvements in shoulder elevation occurred between T1-T3 (p = 0.04, 8–24% of the movement cycle), but were not significant after Bonferroni correction (p > 0.017).

We found a time*group interaction for *scapula tilting* (HTS: p = 0.007, 18–100% of the movement cycle), showing changes over time in the mCIMT + AOT group (p < 0.05, 77–85% of the movement cycle). The increased anterior tilting of the scapula after the intervention (i.e. increased pathological pattern) normalized again at T3 (Fig. 3A).This change was significant only between T2-T3 (cluster 1: p = 0.02, 31–41% of the movement cycle; cluster 2: p = 0.01, 76–100% of the movement cycle). It is important to note that the interaction for this joint angle may have been biased by the increased anterior tilting in the mCIMT + AOT group whilst the mCIMT + placebo group showed decreased anterior tilting (Fig. 3A). Supplementary Fig. S2 (a-l) shows the waveforms at different time points, for each group and for the total group, with additional plotting of normative data from typically developing children, retrieved from Simon-Martinez et al.^25,45^.

Lastly, we also found a time*group interaction for *wrist flexion–extension* (HTS: p < 0.05, 7–17% of the movement cycle, Fig. 3C). Subsequent analysis in each group indicated increased wrist flexion in the mCIMT + placebo group (p < 0.05, 10–16% of the movement cycle).

### Effects of mCIMT (with or without AOT) on kinematic measures

**Figure 4:**
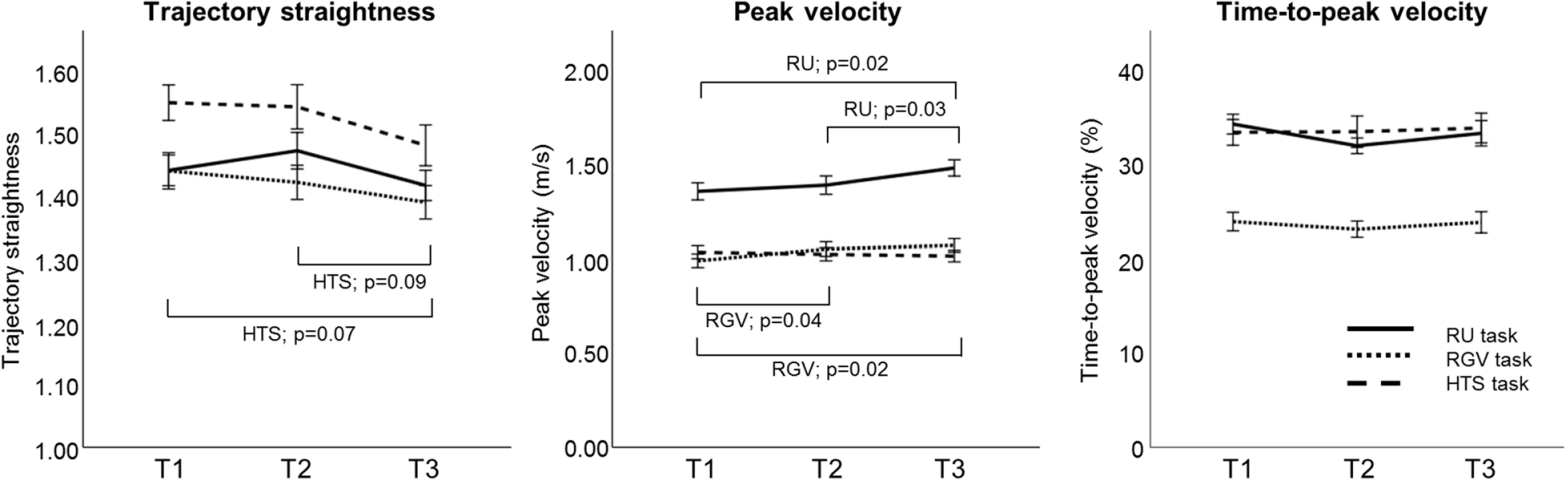
Change in spatiotemporal parameters over time for each task: reach upwards (RU), reach-to-grasp a vertically oriented cylinder (RGV), and hand-to-shoulder (HTS). Lines indicate mean and standard errors.

Both groups together improved over time in *trajectory straightness and peak velocity* (Fig. 4). Trajectory straightness improved during HTS (p = 0.04), and a trend was also found for RU and RGV (p = 0.07) with large effect sizes (partial η^2^ = 0.15 for both tasks, Fig. 4). Post-hoc analyses depicted that these changes occurred between T1-T3 and T2-T3, although they were not significant (p = 0.07, p = 0.09, respectively; Fig. 4). Similar results were found for peak velocity, which significantly changed over time during RU (p = 0.01) and RGV (p = 0.008), also with large effect sizes (partial η^2^ = 0.23–0.25; Fig. 4). Post-hoc analyses for RU showed significant improvements between T1-T3 (p = 0.02) and T2-T3 (p = 0.03). Improvements during RGV were found immediately after the intervention (T1-T2, p = 0.04) and maintained at follow-up (T1-T3, p = 0.02). No improvements over time were found for *time-to-peak velocity* (all tasks, p > 0.05; Fig. 4).

**Figure 5:**
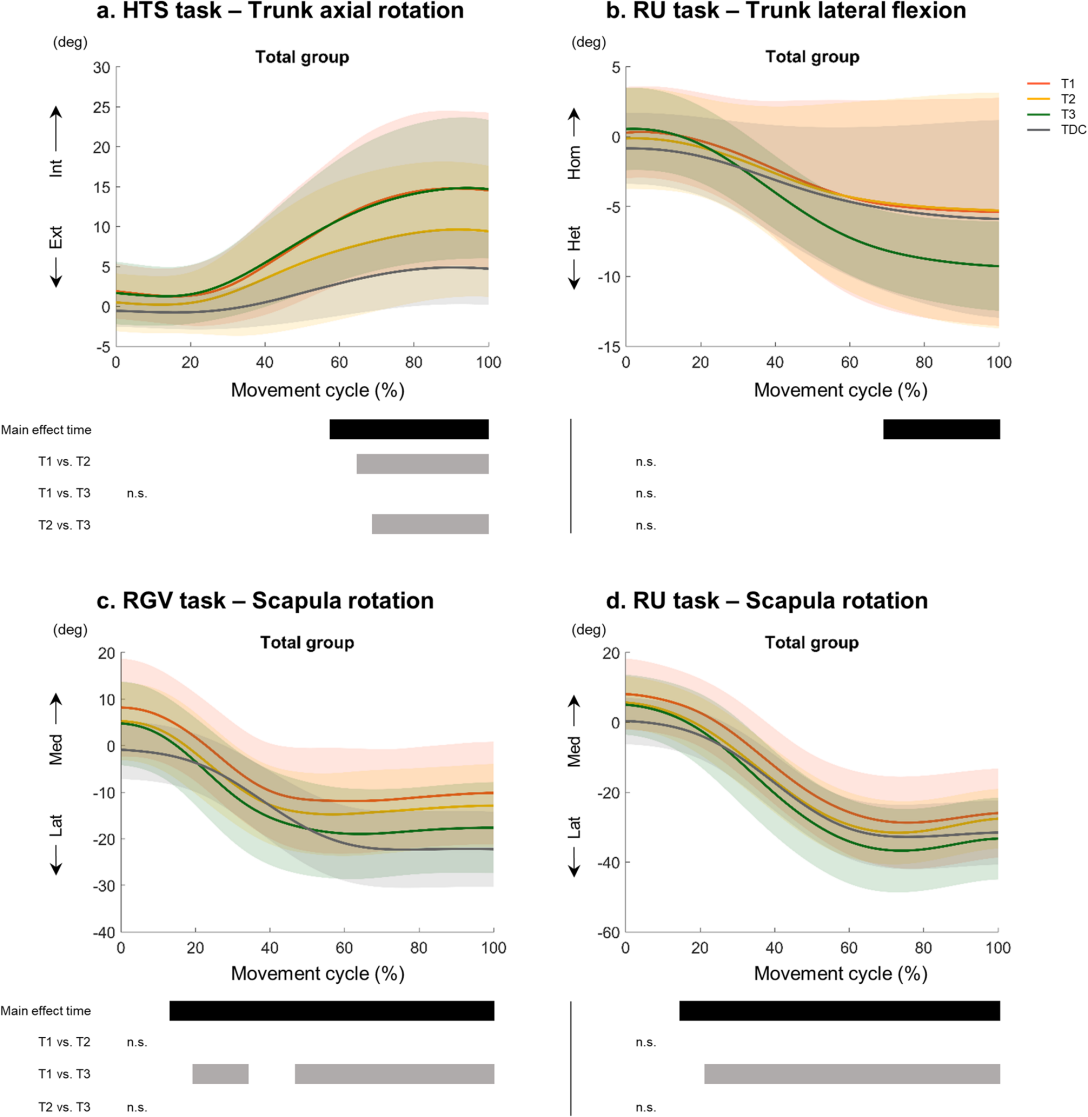
Change in movement pattern for (**A**) trunk axial rotation, (**B**) trunk lateral flexion, and (**C**,**D**) scapula rotation at T1, T2 and T3 for all participants. Mean and standard deviation for the total group is shown. Each subpanel displays where over the waveform the main effects (black bars) and the post-hoc analyses (grey) were depicted. *n.s.* not significant.

A summary of the results of the main effects are reported in Table 3. At the proximal level we found changes in *trunk* axial rotation for HTS (p = 0.02, 58–100% of the movement cycle). Post-hoc analysis showed a trend toward increased external rotation (normalizing the movement pattern) after the intervention (T1–T2, p = 0.03, 64–100% of the movement cycle), which was not maintained at follow up (T1–T3, p > 0.05; T2–T3, p = 0.01, 72–100% of the movement cycle) (Fig. 5A). We also found improved lateral flexion during RU (p = 0.03, 67–100% of the movement cycle), although post-hoc analyses did not survive Bonferroni correction (Fig. 5B).

*Scapula medial–lateral rotation* improved for both reaching tasks (RU: p = 0.002, 15–100% of the movement cycle; RGV: p = 0.002, 14–100% of the movement cycle). Post-hoc analyses showed no differences immediately after the intervention (T1–T2, p > 0.05), but the children used increased lateral rotation at follow-up for RU (T1–T3, p < 0.001, 22–100% of the movement cycle; Fig. 5D) and RGV (T1–T3, cluster 1: p = 0.01, 19–36% of the movement cycle; cluster 2: p = 0.003, 45–100% of the movement cycle; Fig. 5C), resembling the usual movement pattern in typically developing children. There were no changes at the level of the shoulder, elbow or forearm (p > 0.05).

### Relation between improvements in clinical and kinematic measures

The correlation analyses showed a limited association between the improvements in the clinical (muscle tone, muscle weakness and grip strength) and motor control (movement duration, peak velocity, time-to-peak velocity and trajectory straightness) measures. The improvement in trajectory straightness for RU immediately after the camp (T1-T2) showed a low-moderate correlation with decrease in muscle tone (rho = -0.45, p = 0.007, Supplementary Materials Fig. S3), suggesting an association between improvements in trajectory straightness and tone in the flexor UL chain. For the other measures we found no to low correlations between T1 and T2 (Supplementary Materials, Table S2). Between T1 and T3, we found a low-moderate correlation between time-to-peak velocity for HTS and grip strength (rho = − 0.47, p = 0.004, Supplementary Materials Fig. S2), suggesting that an earlier time-to-peak velocity (less time spent in planning the movement) is related to improved grip strength. For other measures, we found no to low correlations between T1 and T3 (Supplementary Materials, Table S3).

## Discussion

This is the first study investigating the effect of an intensive training approach combining mCIMT and AOT on UL motor control and movement patterns in a large cohort of children with uCP. The evaluation included a comprehensive UL kinematic analysis including spatiotemporal parameters and kinematic joint angles, indicative of motor control and movement patterns, respectively. Furthermore, the analysis with SPM1d permitted an investigation on changes in UL movement patterns over the entire waveform. Our results showed that the additional value of AOT was rather small, as measured with 3DMA. The mCIMT + AOT group performed RU significantly faster than the control group, although other spatiotemporal parameters did not change with the intervention. As to UL movement patterns, we found between-groups differences mainly between T2–T3 with inconsistent results between groups. For the total group, we found an improved movement efficiency (peak velocity), a straighter trajectory, and improvements in trunk rotation and lateral flexion as well as in scapula rotation. We did not find changes in movement patterns in the distal joints. These results highlight the importance of using 3DMA to measure changes in proximal joints after an UL intervention.

### What is the added value of AOT to mCIMT on UL kinematics?

The combination of mCIMT + AOT resulted in a shorter movement time with large effect size during one of the three reaching tasks compared to those who did not receive additional AOT. The effect of the video-observation may have paced the rhythm of movement performance, although it is contradicting that a similar result was not found for the other tasks (RGV and HTS). The mean decrease was approximately 7–15% of the total movement time at T1, implying that the clinical relevance may be limited. The only study investigating the effect of AOT on UL kinematics compared to placebo was performed in adult stroke survivors during a period of 6 weeks^46^. In contrast to our study results, they reported improvements in both groups in average velocity and trajectory straightness after the intervention, without between-groups differences in outcomes.

Children who received mCIMT + AOT showed increased anterior scapula tilting after the intervention, which indicated an increased movement pathology^25^. Given that the AOT videos only focused on the hand and the forearm, these increased proximal changes were unexpected and may have occurred at the expense of an improved grasp, as the children may have focused on improving their grasp function potentially leading to proximal compensations. However, grasp function is not assessed with our 3DMA protocol and future studies should include a kinematic evaluation of the grasp after an intervention, together with an evaluation of proximal joints. The abovementioned preliminary study^46^ also pointed toward a limited immediate effect of AOT on UL kinematics in stroke survivors^46^, which is in agreement with our results.

Altogether, our results together with those from the available literature, suggest that AOT may have limited potential in improving UL kinematics (i.e. characteristics of motor control and the quality of the UL movement patterns). There is evidence that children with uCP do improve unimanual and bimanual function after AOT alone^16–20^, linking these improvements to the activation of the mirror neuron system for action understanding, imitation and learning. Nevertheless, there is controversy regarding the role of the mirror neuron system for these aspect^47^ as it is unclear whether action understanding is related to the goal of the action or the motor routine. In our study, children with uCP showed improvements in motor control and movement patterns after mCIMT with a very limited additional effect of AOT, pointing toward a lack of effectiveness in increasing the motor repertoire of children with uCP when AOT is combined with a well-established training approach as mCIMT. Further research in elucidating the mechanisms of AOT in children with uCP is needed to better understand its limitations when combined with other training approaches.

### What are the effects of mCIMT (with or without AOT) on UL kinematics?

After mCIMT, with or without AOT, children generally performed the UL tasks with higher peak velocity, and a straighter trajectory, which may reflect improved motor control, resulting from the intensive skill-based practice in daily life activities. Peak velocity improved in both groups immediately after the intervention, and gains were maintained at follow-up, which is in agreement with previous studies in uCP after mCIMT^48,49^. Children with uCP typically have a lower peak velocity compared to typically developing children^21^, which has been related to muscle strength deficits^24^. The correlation analyses conducted in the current study revealed that the improvements seen in peak velocity are not related to improvements in muscle force. Although this requires further confirmation from future research, our results indicate that the measures of motor control derived from an UL-3DMA provide unique and additional information to the traditional clinical measures, in line with previous research^24^, although they do not seem to be very sensitive to change. Time-to-peak velocity, which represents the movement strategy, did not improve after the intervention. It is known that children with uCP usually do not show impairments in time-to-peak velocity^21,50^, hence little improvement of this parameter was expected. However, it is possible that the tasks that we used were too simple to depict changes in this parameter. Interestingly, we found that the little improvements (between T1 and T3) identified in time-to-peak velocity for the HTS task were related to improvements in grip strength. We also found gains in trajectory straightness, which were more pronounced at follow-up. Our correlation analyses suggested that improvements after the intervention were related to a decrease in muscle tone, although this was not reflected in the other tasks. Robert et al. reported improvements in trajectory straightness immediately after a task-oriented training program of 5 weeks in children with uCP^51^. This may suggest that children with uCP may need more time to improve trajectory straightness and transfer it to their daily life activities. Overall, for all spatiotemporal parameters, it is striking that these improvements were not found across all unimanual tasks and that some tasks seem to be more sensitive to change than others. It could be that the included tasks are not challenging enough to capture overall improvements, but that the evaluation of the different movement phases (reaching, grasping, releasing) may be more sensitive to capture subtle changes in motor control.

Whilst improvements in motor control after mCIMT have been reported, the uniqueness of this study lays within the additional measurement of UL movement patterns with an evaluation at three time points and its analyses over the entire waveform. We found improvements in trunk and scapula movement patterns for both groups combined. More specifically, we found a normalization of the trunk rotation during HTS after mCIMT. Additionally, scapula medial–lateral rotation also normalized during RU and RGV after the intervention (i.e. increased lateral rotation). These proximal improvements may highlight an improved neuromuscular control of the surrounding muscles^52^. Adequate proximal control is crucial for an appropriate use of the distal joints^53^. To the best of our knowledge, changes in joint angles (i.e. movement patterns) have not yet been evaluated after an mCIMT intervention in children with uCP and our results point toward the value of a kinematic evaluation to identify changes in movement patterns after an intensive training.

In contrast to our expectations, we did not find distal improvements after our intervention at the level of the elbow and wrist. Whilst some studies have shown changes at the elbow in children with uCP^54^ and in adult stroke^55–57^, other studies evaluating UL movement quality using e.g. the Melbourne Assessment^58^ have reported no or limited improvements after mCIMT^31,59,60^. These studies highlight the difficulties in changing the distal pathological movement pattern during daily life activities and that a longer training period may be necessary. In our study, we did find training-induced changes at the proximal joints, especially during HTS task, highlighting the responsiveness to change of some 3DMA-derived features related to motor control (i.e. duration, peak velocity, and trajectory straightness). Nevertheless, these small changes should be interpreted with caution and replicated in the future, as their magnitude is small and the implications for function may be limited. More advance versions of the SPM1d software may provide effect sizes, which will be very valuable for the interpretation and generalizability of the findings.

In summary, our study showed some improvements in motor control after mCIMT, with or without AOT, which is in line with the clearly shown benefits for the distal UL measured with traditional clinical scales^61^. However, 3DMA showed its ability and uniqueness in capturing changes in proximal movement patterns. Mailleux et al. previously put forward the importance and advantages of combining both clinical and kinematic UL evaluations, as each contributes in a specific and unique fashion to the identification of UL problems^24^. Integrating both will surely provide a more comprehensive picture of the training-derived changes and will help clinicians target decomposed problems.

There are some limitations in this study. This study’s sample size was calculated to find improvements larger than the smallest detectable difference in bimanual performance (measured with the Assisting Hand Assessment) as a primary outcome measure of the larger study^28^. However, a total sample of 36 children, after counting for missing participant data at T2 and T3, is below the calculated sample size (n = 44) and it may be insufficient to find improvements in distal UL movement patters after a two-week intervention. In this line, the missing participant data may have resulted in a limited generalization of children with uCP, as we excluded children with poor cooperation or who received Botulinum Toxin A. Poor cooperation may be due to the larger time needed to acquire the 3DMA data, whilst the need of Botulinum Toxin A injections resulted in the exclusion of a child with quite limited UL function and high spasticity scores. Therefore, the results of our study should be interpreted considering that some children could not participate due to these clinical characteristics. The improvements in motor control seen in our study could also be observed by using an inertial motion unit, which would reduce costs and burden on the children. Such outside-of-the-lab measurements may also additionally indicate whether the improvements have functional implications in terms of amount of use or increase of active range of motion^62,63^. Nevertheless, 3DMA has been shown to be useful in providing unique and additional information about UL kinematics after joint-specific treatments, like Botulinum Toxin A and surgery^26,27^. A second limitation of this study relates to the UL-3DMA protocol, as it did not measure grasp aperture and closure. Other 3DMA protocols measuring grasp and finger movements^49,64^ might be able to capture improvements at these levels. Finally, for both the spatiotemporal characteristics and the joint angles, we found large individual variability in response to the intervention. Previous studies have also shown large variation in clinical outcome measures after an mCIMT intervention^65^, suggesting that there might be sub-groups of children who respond better. Although this has not yet been explored with a kinematic outcome, further analysis could also focus on identifying responders based on movement characteristics.

Future studies could also include bimanual tasks to evaluate improvements in bimanual coordination and the transfer of skills from a unimanual to a bimanual level, which has received very little attention in research^66^. The use of SPM1d in this study has shown its ability to map treatment-dependent changes in uCP and could be included also in studies including kinematics during bimanual tasks. In addition to bimanual coordination, it would be interesting to include muscle activity measures (i.e. electromyography), which will allow for a more comprehensive understanding of the changes at the muscle activity level. In this line, the investigation of muscle synergies has been shown to offer insights into the complexity of motor control in uCP^67,68^.

In conclusion, adding AOT to mCIMT affects movement duration during reaching, whereas limited benefit is observed on movement patterns. However, independent of AOT, mCIMT improved characteristics of motor control and proximal movement patterns, which are important to stabilize the UL to exert a coordinated, effective and smooth movement. This study adds to the available literature on intensive therapies in uCP and highlights the importance of including 3DMA in the UL evaluation to capture changes in motor control at proximal joints. Furthermore, we contributed to the identification of specific outcome parameters and tasks sensitive to change, advancing the clinical implementation of UL-3DMA.

## Supplementary information

Supplementary information 1.

Supplementary information 2. 

Supplementary information 3. 

Supplementary information 4. 

## Data Availability

Data related to the manuscript is available upon request to corresponding author.
