# Supplementary material for: Effects of combining constraint-induced movement therapy and action-observation training on upper limb kinematics in children with unilateral cerebral palsy: a randomized controlled trial
Source: Sci Rep. 2020 Jun 26;10:10421. doi: 10.1038/s41598-020-67427-2 (PMC7320002; doi:10.1038/s41598-020-67427-2)
Supplement: Supplementary file 1 — Supplementary information 1. [file 41598_2020_67427_MOESM1_ESM.pdf]

ClinicalTrials.gov Protocol Registration and Results System (PRS) Receipt
Release Date: October 25, 2019

ClinicalTrials.gov ID: NCT03256357

Study Identification

Unique Protocol ID: CIMT and AOT in unilateral CP

Brief Title: Constraint-Induced Movement Therapy and Action Observation Training in Children With Unilateral Cerebral Palsy

Official Title: Effectiveness of Intensive Upper Limb Training Combining Constraint-Induced Movement Therapy and Action Observation Training in Children With Unilateral Cerebral Palsy

Secondary IDs:

Study Status

Record Verification: October 2019

Overall Status: Completed

Study Start: January 1, 2014 [Actual]

Primary Completion: February 28, 2018 [Actual]

Study Completion: February 28, 2018 [Actual]

Sponsor/Collaborators

Sponsor: KU Leuven

Responsible Party: Principal Investigator
Investigator: Klingels Katrijn [kkatrijn]
Official Title: Principal Investigator
Affiliation: KU Leuven

Collaborators: ETH Zurich (Switzerland)
University of Pisa, Italy

Oversight

U.S. FDA-regulated Drug:

U.S. FDA-regulated Device:

U.S. FDA IND/IDE: No

Human Subjects Review: Board Status: Approved
Approval Number: S56513
Board Name: Ethical Committee of the university hospitals Leuven
Board Affiliation: University Hospitals Leuven
Phone: 003216348600
Email: ec@uzleuven.be

Address:

Data Monitoring:

Study Description

Brief Summary: A randomized, controlled, and evaluator-blinded trail will be carried out comparing CIMT with or without AOT on sensorimotor outcome in children with unilateral CP aged 5 to 12 years. Additionally the potential role of neurological factors, including the anatomical characterization of the brain lesion, structural/ functional connectivity and cortical reorganization, on treatment response will be investigated.

Detailed Description: Background: Problems in upper limb (UL) function in children with unilateral cerebral palsy (UCP) are traditionally trained with motor execution treatment models, such as Constraint Induced Movement Therapy (CIMT). However new approaches based on a neurophysiological model such as action observation training (AOT) may provide new opportunities for enhanced motor learning.

Aim: The aim of study is to investigate the effects of an intensive treatment model consisting of CIMT and AOT compared to CIMT alone on UL function in children with UCP. Additionally the potential role of neurological factors (including the anatomical characterization of the brain lesion, structural/ functional connectivity and cortical reorganization) on treatment response will be analysed.

Methods/Design: A randomized, controlled, evaluator-blinded trial (RCT) will be conducted in 40 children between 5 and 12 years of age. Before randomization, children are stratified according to their House Functional Classification Scale, age and type of cortical reorganization. The Intervention is accomplished during a 2-week day camp in which the children receive intensive therapy for six hours a day on 9 out of 11 consecutive days (54 h) including AOT or placebo observation training (POT) (15h). During the AOT the children in the experimental group watch video sequences showing goal-directed actions and subsequently execute the observed actions with the affected UL. Children in the POT group perform the same actions after watching computer games without biological movements.

Outcome assessments include qualitative and quantitative measures of UL sensorimotor function across the International Classification of Functioning, Disability and Health (ICF). The primary outcome measure is the Assisting Hand Assessment (AHA). The medical imaging protocol includes structural Magnetic Resonance Imaging (MRI), Diffusion Kurtosis Imaging (DKI), resting state functional MRI (rs-fMRI) and Transcranial magnetic stimulation (TMS). The timeline for the assessment is T0 (1-1.5 month before the camp onset), T1 (before the intervention), T2 (after the intervention) and T3 (6 months after the intervention). Linear mixed models will be used to study time effects of the interventions and the interaction with neurological variables as covariates.

Conditions

Conditions: Cerebral Palsy

Keywords: unilateral cerebral palsy
upper limb rehabilitation

Study Design

Study Type:	Interventional
Primary Purpose:	Treatment
Study Phase:	N/A
Interventional Study Model:	Parallel Assignment
Number of Arms:	2
Masking:	Double (Participant, Outcomes Assessor)
Allocation:	Randomized
Enrollment:	44 [Actual]

Arms and Interventions

Arms	Assigned Interventions
Active Comparator: CIMT + AOT<br>In a 2-week day camp model children receive constraint-induced movement therapy for six hours a day, for 9 out of 11 consecutive days. All children wear a tailor made hand splint on the unaffected upper limb for 6 hours per day while performing unimanual exercises individually or in a group based on shaping and repetitive practice.<br><br>Action observation training consists of 15 sessions of 1 hour. Children watch video sequences showing goal-directed actions and subsequently execute the observed actions with the affected upper limb.	Behavioral: Constraint-induced movement therapy<br>In a 2-week day camp model children receive constraint- induced movement therapy for six hours a day, for 9 out of 11 consecutive days. All children wear a tailor made hand splint on the unaffected upper limb for 6 hours per day while performing unimanual exercises individually or in a group based on shaping and repetitive practice.<br>Behavioral: Action observation training<br>Action observation training consists of 15 sessions of 1 hour. Children watch 3 minute video clips of unimanual goal-directed actions followed by 3 minutes of execution of the actions.
Placebo Comparator: CIMT + POT<br>In a 2-week day camp model children receive constraint- induced movement therapy for six hours a day, for 9 out of 11 consecutive days. All children wear a tailor made hand splint on the unaffected upper limb for 6 hours per day while performing unimanual exercises individually or in a group based on shaping and repetitive practice.<br><br>Placebo observation training consists of 15 sessions of 1 hour.This group performs the same actions after watching computer games without biological movements.	Behavioral: Constraint-induced movement therapy<br>In a 2-week day camp model children receive constraint- induced movement therapy for six hours a day, for 9 out of 11 consecutive days. All children wear a tailor made hand splint on the unaffected upper limb for 6 hours per day while performing unimanual exercises individually or in a group based on shaping and repetitive practice.<br>Behavioral: Placebo observation training<br>Placebo observation training consists of 15 sessions of 1 hour. Children perform the same actions as the AOT training after watching computer games without biological movements.

Outcome Measures

Primary Outcome Measure:

1. Change in Assisting Hand Assessment (AHA)
   The AHA, a Rasch-based performance scale, measures how effectively the affected hand is spontaneously used during performance of bimanual tasks.

   [Time Frame: baseline, pre and post intervention (within one week), 6 months follow-up]

Secondary Outcome Measure:

2. Change in Melbourne Assessment of Unilateral Upper Limb Function
   The Melbourne Assessment evaluates quality of movement in 16 functional unimanual tasks.

[Time Frame: baseline, pre and post intervention (within one week), 6 months follow-up]

3. Change in Jebsen-Taylor Hand Function Test
   The Jebsen-Taylor hand function test measures manual dexterity in six unimanual tasks, by means of movement time expressed in seconds for both hands.

   [Time Frame: baseline, pre and post intervention (within one week), 6 months follow-up]

4. Change in Tyneside Pegboard test
   The Tyneside pegboard test is an adapted 9-hole pegboard test and assesses unimanual and bimanual dexterity.

   [Time Frame: pre and post intervention (within one week), 6 months follow-up]

5. Change in passive range of motion (PROM)
   PROM of shoulder flexion, abduction, external and internal rotation, elbow extension, forearm supination and wrist extension is measured using a universal goniometer.

   [Time Frame: baseline, pre and post intervention (within one week), 6 months follow-up]

6. Change in muscle tone
   Muscle tone is evaluated in 11 muscle groups using the Modified Ashworth Scale (MAS), ranging from 0 to 4.

   [Time Frame: baseline, pre and post intervention (within one week), 6 months follow-up]

7. Change in muscle strength
   Muscle strength is evaluated in nine muscle groups using manual muscle testing (MMT), ranging from 0 to 5.

   [Time Frame: baseline, pre and post intervention (within one week), 6 months follow-up]

8. Change in grip strength
   Grip strength is assessed with a Jamar dynamometer®. The average of three consecutive maximum contractions is recorded for both hands.

   [Time Frame: baseline, pre and post intervention (within one week), 6 months follow-up]

9. Change in muscle fatigability
   Muscle fatigability during an isometric grip strength task is assessed based on a 30 second sustained contraction with E-link software.

   [Time Frame: baseline, pre and post intervention (within one week), 6 months follow-up]

10. Change in Abilhand-Kids questionnaire
    The Abilhand- Kids questionnaire is a Rasch-based inventory of 21 mostly bimanual activities that the parents are asked to judge as: 0 (impossible), 1 (difficult), and 2 (easy), irrespective of the limb(s) actually used to do the activity.

    [Time Frame: baseline, pre and post intervention (within one week), 6 months follow-up]

11. Change in Children's Hand-use Experience Questionnaire (CHEQ)
    CHEQ is a questionnaire to evaluate the experience of children and adolescents in using the affected hand in activities where usually two hands are needed.

    [Time Frame: baseline, pre and post intervention (within one week), 6 months follow-up]

12. Change in Upper limb Three-dimensional movement analysis (3DMA)
    This quantitative assessment comprises upper limb kinematics during functionally relevant aiming and grasping tasks.

    [Time Frame: pre and post intervention (within one week), 6 months follow-up]

13. Change in Assessment of Life Habits for children (LIFE-H Kids)
    LIFE-H Kids assesses the quality of social participation of children with disabilities by estimating how a client accomplishes activities of daily living and social roles

    [Time Frame: pre intervention (within one week), 6 months follow-up]

14. Change in Cerebral Palsy Quality of Life Questionnaire (CP-QOL)
    CP-QOL is a parent report that assesses the wellbeing across various domains of life in children with cerebral palsy.

    [Time Frame: pre intervention (within one week), 6 months follow-up]

Other Pre-specified Outcome Measures:

15. Structural Magnetic Resonance Imaging (MRI)
Structural images are acquired using three-dimensional fluid-attenuated inversion recovery (3D FLAIR) with following parameters: 321 sagittal slices, slice thickness 1.2, slice gap 0.6, repetition time=4800 ms, echo time=353 ms, field of view=250 x 250 mm², 1.1 x 1.1 x 0.56 mm³ voxel size, acq time = 5’02”. Brain lesions will be first classified according to the timing of the lesion and the predominant pattern of damage as described by Kragelöh-mann (2007): cortical malformations (first and second trimester of pregnancy), periventricular white matter (PWM) lesions (from late second till early third trimester) and cortical and deep greymatter (CDGM) lesions (around term age) and acquired brain lesions (between 28 days 3 years postnatally). Second, a more detailed evaluation of the brain lesion (i.e. location and extent) will be performed using the semi-quantitative MRI (sqMRI) scale developed by Fiori et al. (2014).

[Time Frame: baseline]

16. Resting state functional Magnetic Resonance Imaging (rsfMRI)
rsfMRI images are acquired using a T2*-weighted gradient-echo planar imaging (GE-EPI) sequence with the following parameters: TR = 1700 ms; TE = 30 ms; matrix size = 64x64; FOV = 230 mm; flip angle = 90º; slice thickness = 4 mm; no gap; axial slices = 30; number of functional volumes = 250; acquisition time = 7 min. rsfMRI will be pre-processed with Statistical Parametric Mapping version 12 (SPM12) software. Functional connectivity analysis will be computed with the CONN toolbox v17b.

[Time Frame: baseline]

17. Diffusion weighted imaging
Diffusion weighted images will be acquired using a single shot spin echo sequence with the following parameters: slice thickness = 2.5 mm, TR = 8700 ms, TE = 116 ms, number of diffusion directions = 150, number of sagittal slices = 58, voxel size = 2.5 x 2.5 x 2.5 mm³, acq time = 18’. Implemented b values are 700, 1000, and 2800 s/mm², applied in 25, 40, and 75 uniformly distributed directions, respectively. In addition, 11 non-diffusion weighted images are obtained. Diffusion data will be pre-processed and analyzed in ExploreDTI toolbox, version 4.8.6. Diffusion metrics, such as fractional anisotropy and mean diffusivity of white matter tracts of interest (i.e. corpus callosum, corticospinal tract, superior thalamic radiations, medial lemniscus) will be calculated for both hemispheres.

[Time Frame: baseline]

18. Transcranial Magnetic Stimulation (TMS)
TMS was performed using a MagStim 200 Stimulator (Magstim Ltd, Whitland, Wales, UK) equipped with a focal 70mm figure-eight coil and a Bagnoli electromyography (EMG) system with two single differential surface electrodes (Delsys Inc, Natick, MA, USA). A Micro1401-3 acquisition unit and Spike software version 4.11 (Cambridge Electronic Design Limited, Cambridge, UK) were used to synchronize the TMS stimuli and the EMG data acquisition. Motor Evoked Potentials (MEPs) were bilaterally recorded, using single differential surface EMG electrodes attached on the muscles adductor pollicis brevis of both hands.

[Time Frame: baseline]

Eligibility

Minimum Age: 5 Years

Maximum Age: 12 Years

Sex: All

Gender Based: No

Accepts Healthy Volunteers: No

Criteria: Inclusion Criteria:

- confirmed diagnosis of unilateral CP
- aged 5-12 years
- sufficient cooperation to comprehend and complete the test procedure
- minimal ability to actively grasp and stabilize an object

Exclusion Criteria:

- upper limb surgery two years prior to enrollment

• botulinum toxin A injections six months prior to enrollment

Contacts/Locations

Central Contact Person: Katrijn Klingels, Dr
Email: katrijn.klingels@kuleuven.be

Central Contact Backup:

Study Officials: Cristina Simon-Martinez
Study Principal Investigator
KU Leuven

Hilde Feys, Prof
Study Director
KU Leuven

Locations: **Belgium**
KU Leuven
Leuven, Belgium, 3000
Contact: Katrijn Klingels

IPDSharing

Plan to Share IPD: Undecided

References

Citations:

Links:

Available IPD/Information:
