# Supplementary material for: Effects of combining constraint-induced movement therapy and action-observation training on upper limb kinematics in children with unilateral cerebral palsy: a randomized controlled trial
Source: Sci Rep. 2020 Jun 26;10:10421. doi: 10.1038/s41598-020-67427-2 (PMC7320002; doi:10.1038/s41598-020-67427-2)

**Effects of combining constraint-induced movement therapy and action-observation training on upper limb kinematics in children with unilateral cerebral palsy: A randomized controlled trial.**

Cristina Simon-Martinez^1,2^*, [cristina.simon@kuleuven.be](mailto:cristina.simon@kuleuven.be)

Lisa Mailleux^1^, [lisa.mailleux@kuleuven.be](mailto:lisa.mailleux@kuleuven.be)

Ellen Jaspers^3^, [ellen.jaspers@hocoma.com](mailto:ellen.jaspers@hocoma.com)

Els Ortibus^4^, [els.ortibus@uzleuven.be](mailto:els.ortibus@uzleuven.be)

Kaat Desloovere^1,5^, [kaat.desloovere@uzleuven.be](mailto:kaat.desloovere@uzleuven.be)

Katrijn Klingels^1,6^, [katrijn.klingels@kuleuven.be](mailto:katrijn.klingels@kuleuven.be)

Hilde Feys^1^, [hilde.feys@kuleuven.be](mailto:hilde.feys@kuleuven.be)

^1^ KU Leuven, Department of Rehabilitation Sciences, Leuven, Belgium.

^2^ Information Systems Institute, University of Applied Sciences Western Switzerland Valais (HES-SO Valais) Sierre, Switzerland.

^3^ Neural Control of Movement Lab, ETH Zurich, Zurich, Switzerland.

^4^ KU Leuven, Department of Development and Regeneration, Leuven, Belgium.

^5^ University Hospitals Leuven, Clinical Motion Analysis Laboratory, Leuven, Belgium

^6^ Rehabilitation Research Centre, Faculty of Rehabilitation Sciences, Hasselt University, Diepenbeek, Belgium.

*Correspondence:

Cristina Simon-Martinez, [cristina.simon@kuleuven.be](mailto:cristina.simon@kuleuven.be)

Herestraat 49, box 1510

3000 Leuven

## Supplementary Materials

**Table S1.** Descriptive characteristics of the participants per group and statistical comparison.

|  |  | **mCIMT+AOT (n=20)°** | **mCIMT+placebo (n=16)*** | **p-value** |
| --- | --- | --- | --- | --- |
| **Age** | Mean (line) (SD) | 9y4m (1y11m) | 9y6m (1y10m) | 0.76^a^ |
| **Sex** | n (%) |  |  | 0.11^b^ |
| Boys |  | 14 (70) | 7 (44) |  |
| Girls |  | 6 (30) | 9 (56) |  |
| **Affected side** | n (%) |  |  |  |
| Left |  | 7 (35) | 11 (69) |  |
| Right |  | 13 (65) | 5 (31) |  |
| **MACS** | n (%) |  |  | 0.90^b^ |
| I |  | 5 (25) | 3 (19) |  |
| II |  | 7 (35) | 6 (37) |  |
| III |  | 8 (40) | 7 (44) |  |
| **HFC system** | n (%) |  |  | 0.65^b^ |
| Levels 4-5 |  | 15 (65) | 13 (81) |  |
| Level 6-8 |  | 5 (35) | 3 (19) |  |

MACS, Manual Ability Classification System; HFC, House Functional Classification. ^a^Mann-Whitney U Test; ^b^Chi-square test. °Missing participant data after the intervention (n=2); *Missing participant data after intervention (n=4) and at follow up (n=2).

**Table S2.** Correlation analysis results (Spearman’s rho and p-value) between improvements in spatiotemporal parameters and clinical measures immediately after the camp (T1-T2).

|  |  |  | **Muscle tone** | **Muscle weakness** | **Grip strength** |
| --- | --- | --- | --- | --- | --- |
| **Reaching upward (RU)** | | |  |  |  |
|  | Duration | Spearman’s rho (p) | -0.13 (0.46) | 0.10 (0.54) | 0.14 (0.42) |
|  | Peak velocity | Spearman’s rho (p) | 0.04 (0.82) | 0.14 (0.40) | -0.10 (0.58) |
|  | Time to peak velocity | Spearman’s rho (p) | -0.18 (0.29) | -0.13 (0.44) | 0.15 (0.38) |
|  | Trajectory straightness | Spearman’s rho (p) | **-0.45 (0.007)** | 0.26 (0.13) | 0.11 (0.53) |
| **Reach-to-grasp a vertically oriented cylinder (RGV)** | | | |  |  |
|  | Duration | Spearman’s rho (p) | 0.00 (0.99) | 0.01 (0.95) | -0.06 (0.71) |
|  | Peak velocity | Spearman’s rho (p) | -0.11 (0.53) | -0.03 (0.87) | 0.09 (0.59) |
|  | Time to peak velocity | Spearman’s rho (p) | -0.34 (0.05) | -0.13 (0.46) | 0.10 (0.55) |
|  | Trajectory straightness | Spearman’s rho (p) | 0.19 (0.27) | -0.35 (0.04) | -0.10 (0.56) |
| **Hand to shoulder (HTS)** | | |  |  |  |
|  | Duration | Spearman’s rho (p) | -0.18 (0.29) | -0.13 (0.44) | 0.15 (0.38) |
|  | Peak velocity | Spearman’s rho (p) | 0.25 (0.16) | 0.16 (0.36) | -0.14 (0.40) |
|  | Time to peak velocity | Spearman’s rho (p) | 0.27 (0.11) | -0.19 (0.27) | -0.19 (0.26) |
|  | Trajectory straightness | Spearman’s rho (p) | -0.13 (0.47) | -0.04 (0.83) | 0.21 (0.22) |

*The correlations that reached p<0.01 significance are highlighted in* ***bold.***

**Table S3.** Correlation analysis results (Spearman’s rho and p-value) between improvements in spatiotemporal parameters and clinical measures immediately after the camp (T1-T3).

|  |  |  | **Muscle tone** | **Muscle weakness** | **Grip strength** |
| --- | --- | --- | --- | --- | --- |
| **Reaching upward (RU)** | | |  |  |  |
|  | Duration | Spearman’s rho (p) | -0.06 (0.75) | 0.00 (0.99) | -0.03 (0.88) |
|  | Peak velocity | Spearman’s rho (p) | 0.33 (0.05) | 0.15 (0.37) | 0.06 (0.72) |
|  | Time to peak velocity | Spearman’s rho (p) | -0.23 (0.19) | -0.08 (0.64) | -0.14 (0.43) |
|  | Trajectory straightness | Spearman’s rho (p) | 0.09 (0.61) | 0.33 (0.05) | 0.13 (0.45) |
| **Reach-to-grasp a vertically oriented cylinder (RGV)** | | | |  |  |
|  | Duration | Spearman’s rho (p) | -0.30 (0.08) | -0.14 (0.43) | -0.10 (0.56) |
|  | Peak velocity | Spearman’s rho (p) | 0.27 (0.11) | 0.11 (0.53) | 0.07 (0.69) |
|  | Time to peak velocity | Spearman’s rho (p) | -0.06 (0.75) | 0.06 (0.72) | 0.01 (0.95) |
|  | Trajectory straightness | Spearman’s rho (p) | 0.00 (0.98) | -0.28 (0.10) | 0.00 (0.98) |
| **Hand to shoulder (HTS)** | | |  |  |  |
|  | Duration | Spearman’s rho (p) | -0.41 (0.01) | -0.06 (0.73) | 0.22 (0.19) |
|  | Peak velocity | Spearman’s rho (p) | 0.25 (0.14) | 0.08 (0.63) | 0.04 (0.80) |
|  | Time to peak velocity | Spearman’s rho (p) | -0.06 (0.73) | -0.25 (0.14) | **-0.47 (0.004)** |
|  | Trajectory straightness | Spearman’s rho (p) | -0.39 (0.02) | -0.05 (0.76) | -0.08 (0.66) |

*The correlations that reached p<0.01 significance are highlighted in* ***bold.***

**Figure S1.** Marker locations at the start (left panels) and end (right panels) position of each task: (a) reaching forward, (b) reach-to-grasp a vertically oriented cylinder and (c) hand to shoulder. This figure was created using Vicon Nexus software (version 1.8.5, Oxford Metrics, Oxford, UK, <https://www.vicon.com/software/nexus/?section=downloads>).


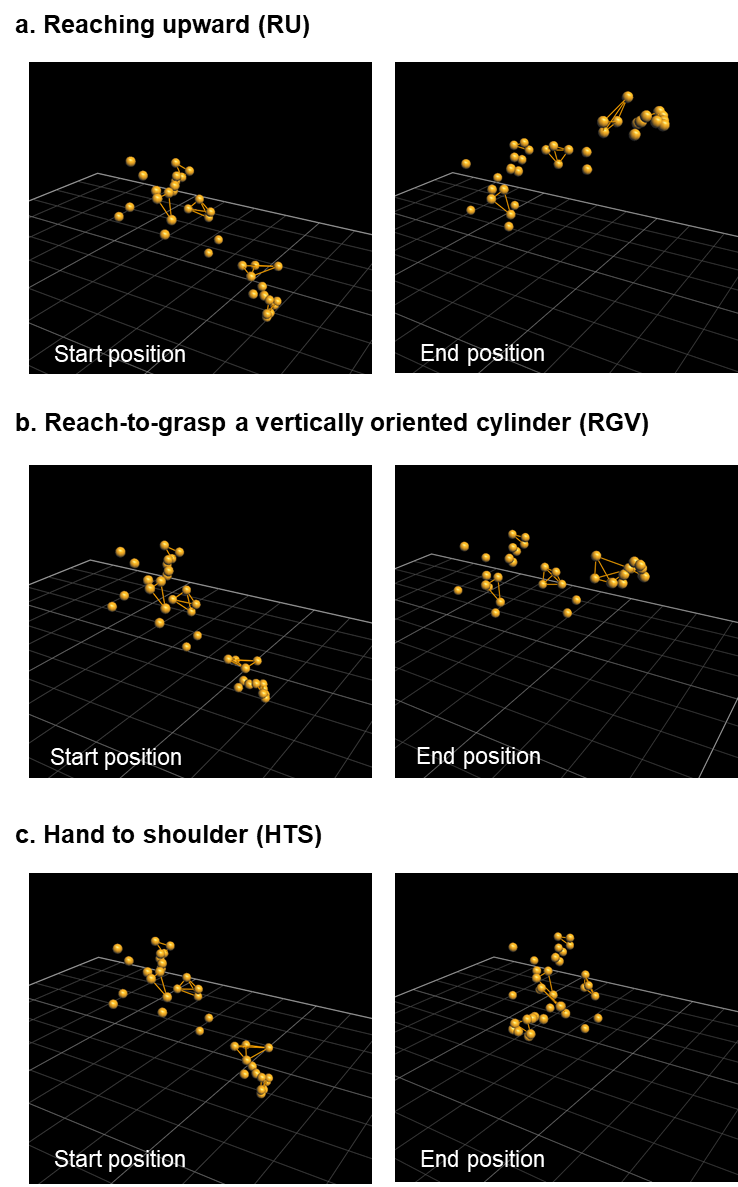


**Figure S2.** Mean (line) and standard deviation (translucent area) of each joint angle at each time point for the mCIMT+AOT group (left), the mCIMT+placebo group (middle), and the total group (right). Each panel represents one task. The grey line depicts the mean (line) and standard deviation (translucent area) of a group of 60 typically developing children aged 5-15y, which is plotted for visualization of a normal movement pattern. (a) trunk flexion-extension, (b) trunk lateral flexion, (c) trunk axial rotation, (d) scapula tilting, (e) scapula pro-retraction, (f) scapula rotation, (g) shoulder elevation, (h) shoulder rotation, (i) shoulder elevation plane, (j) elbow flexion-extension, (k) elbow pro-supination, and (l) wrist flexion-extension.


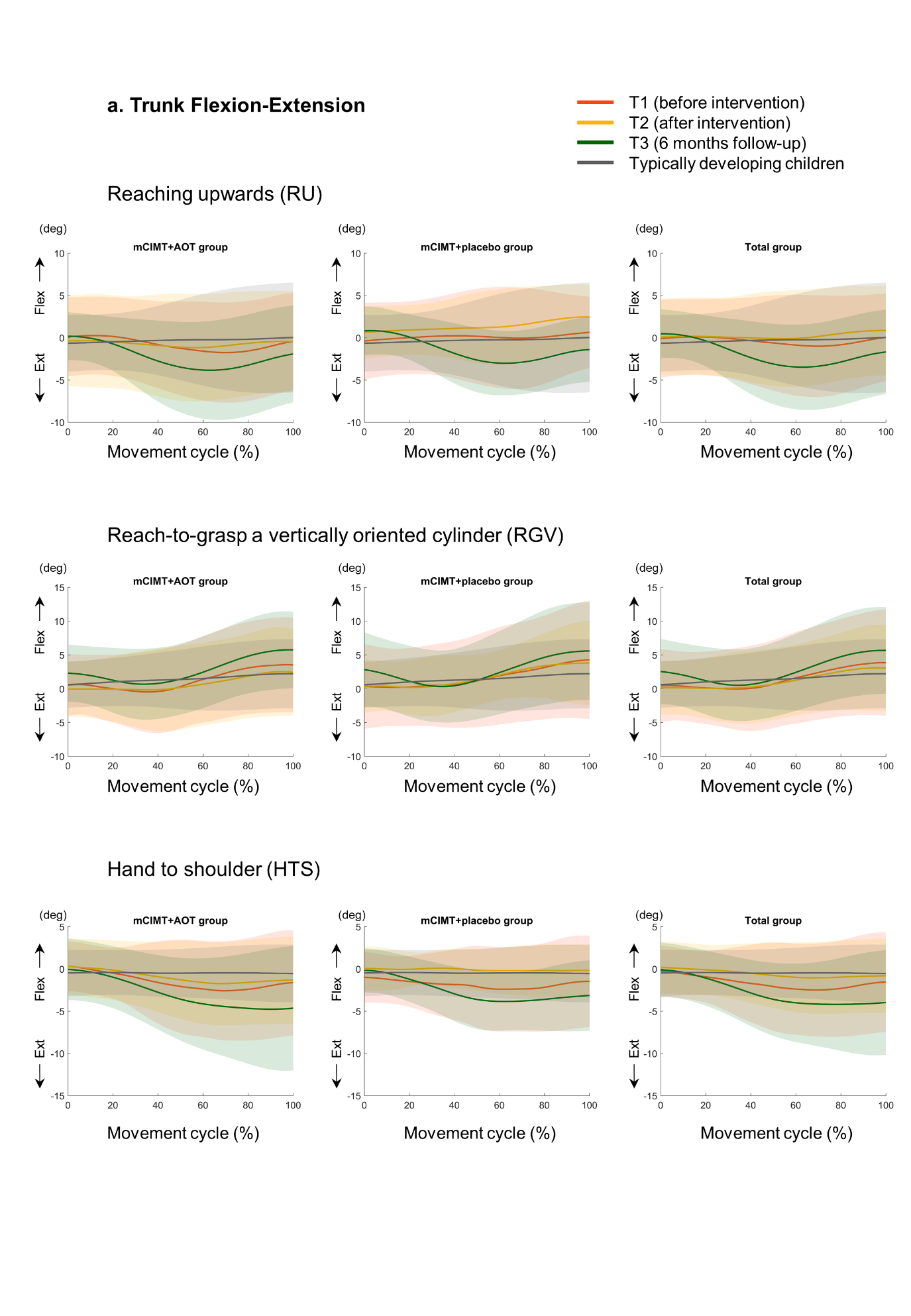


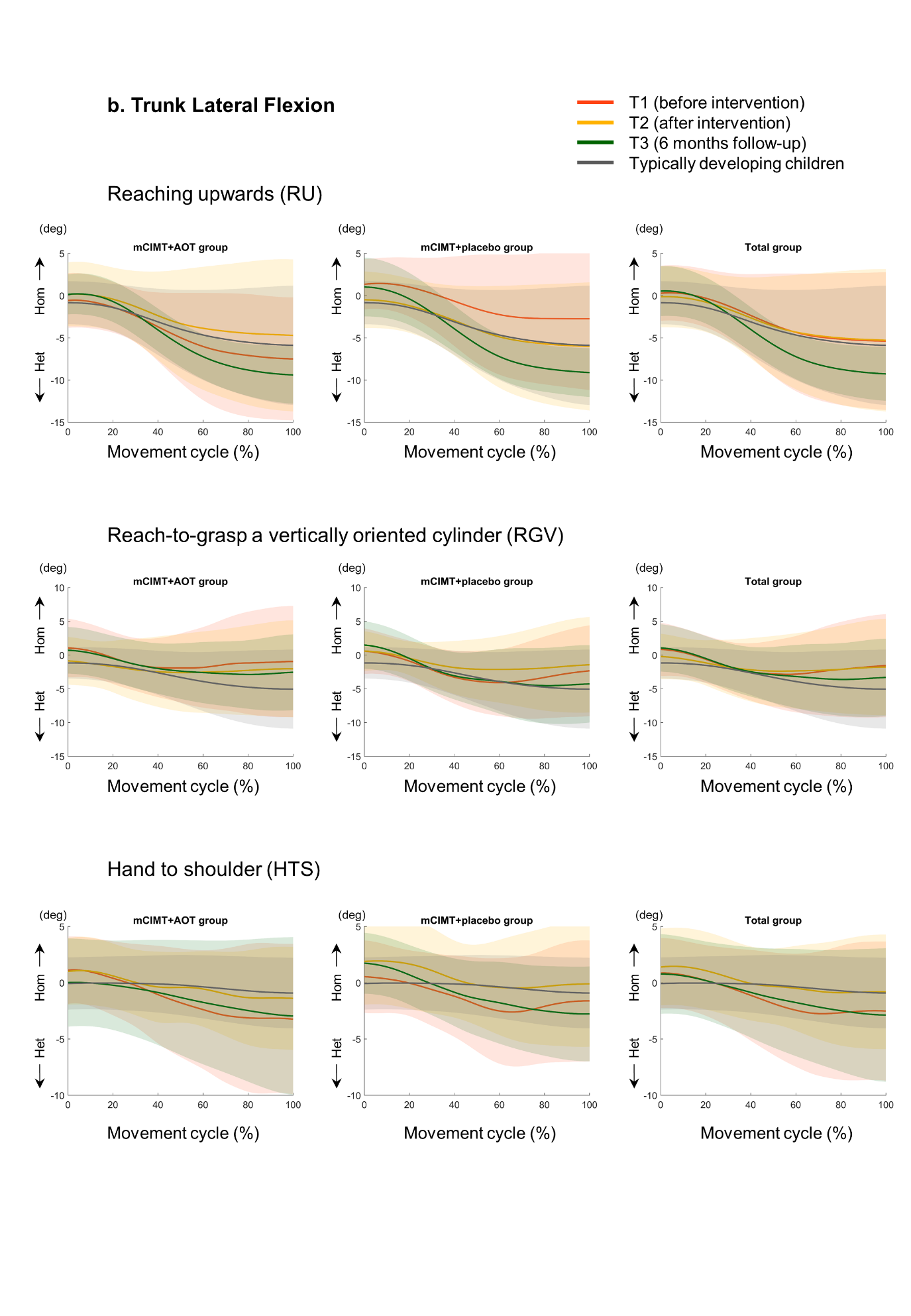


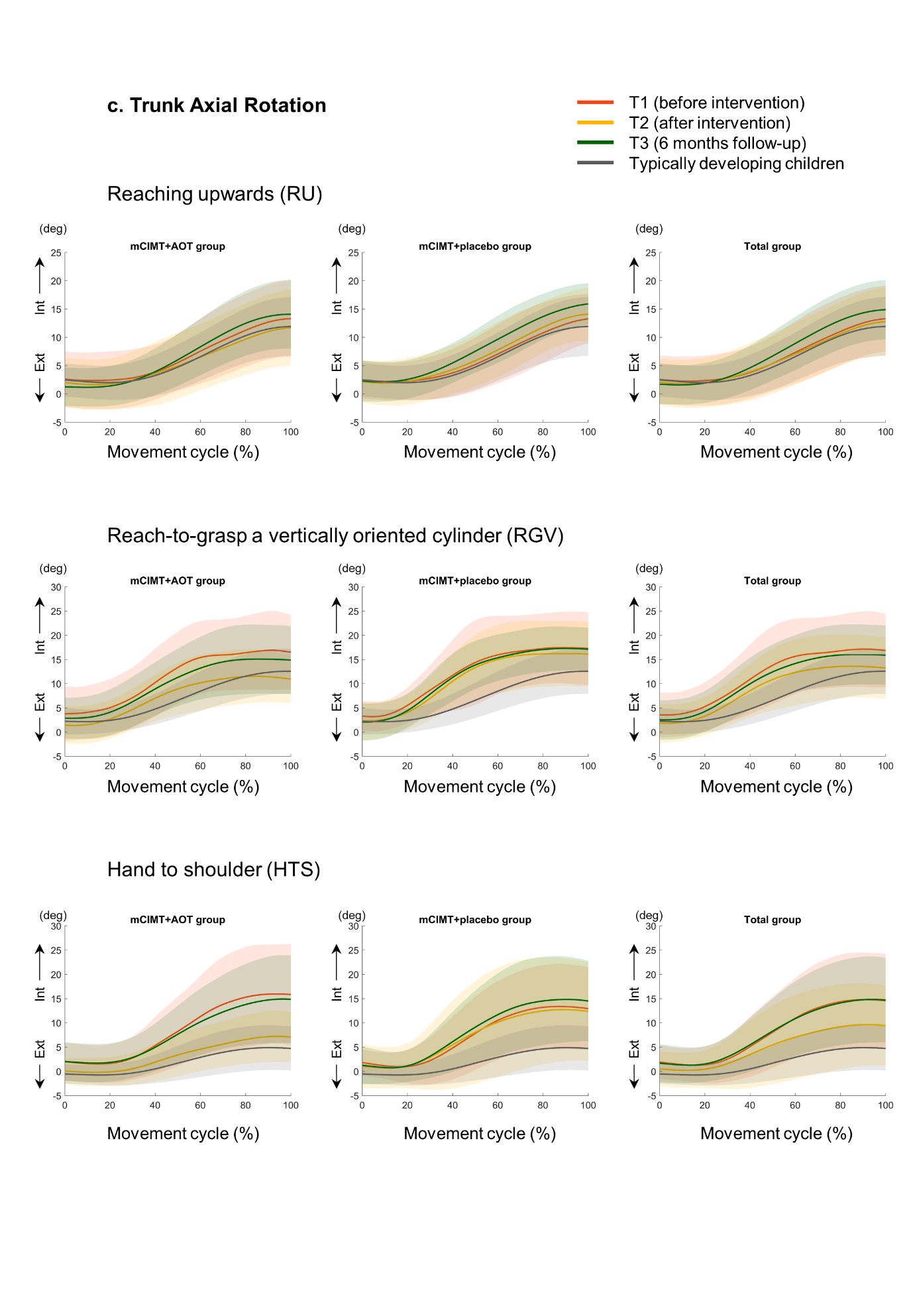


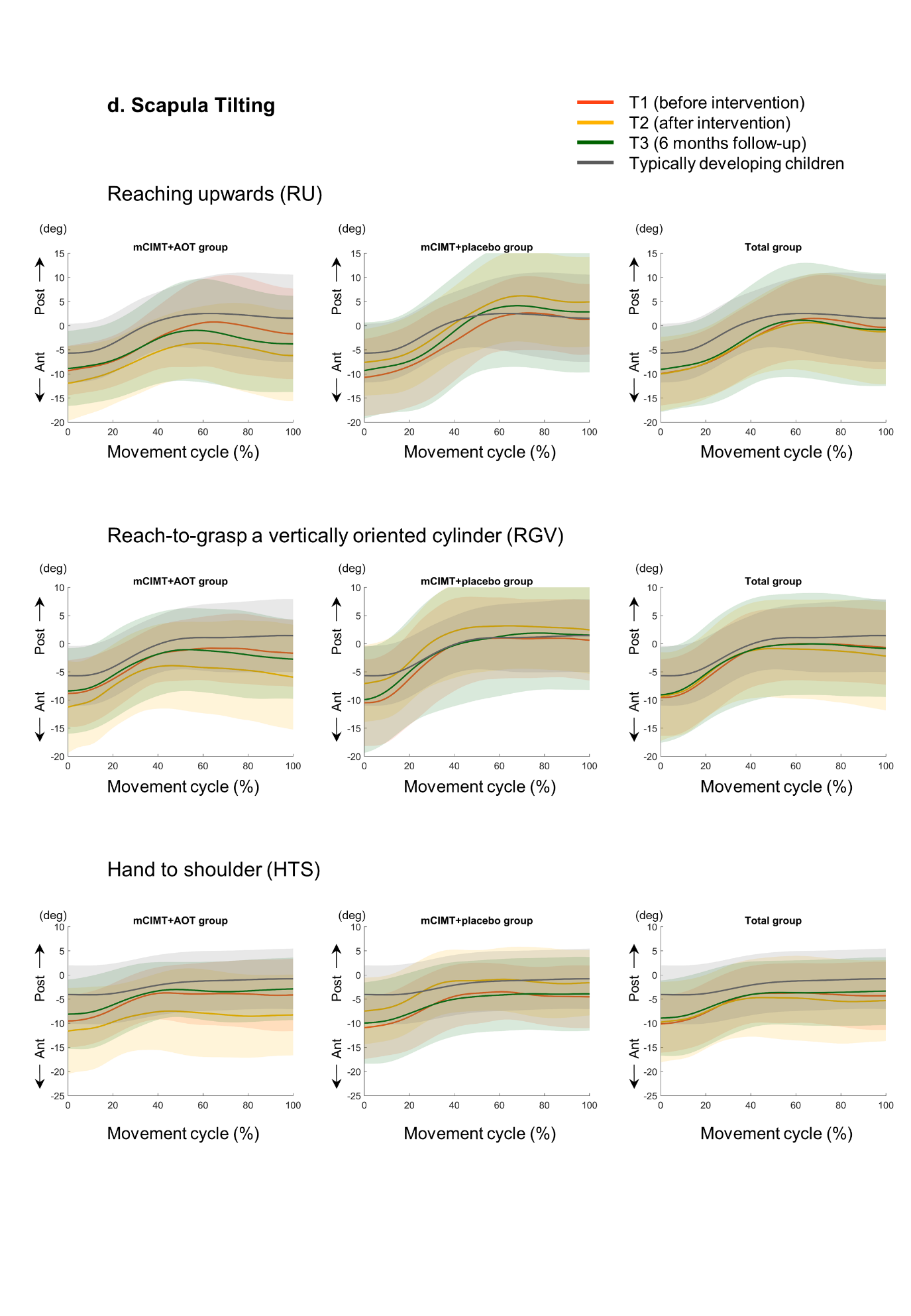


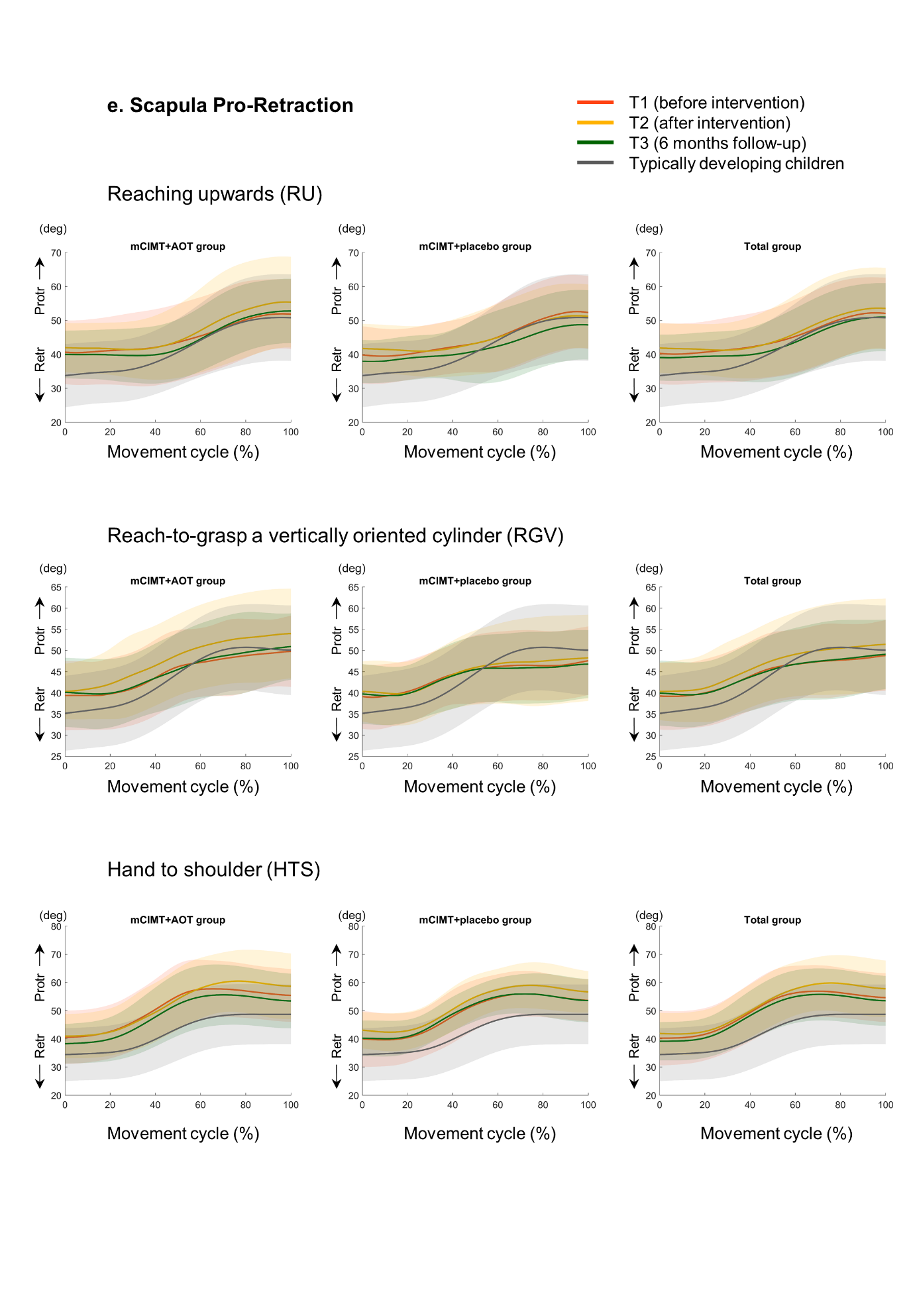


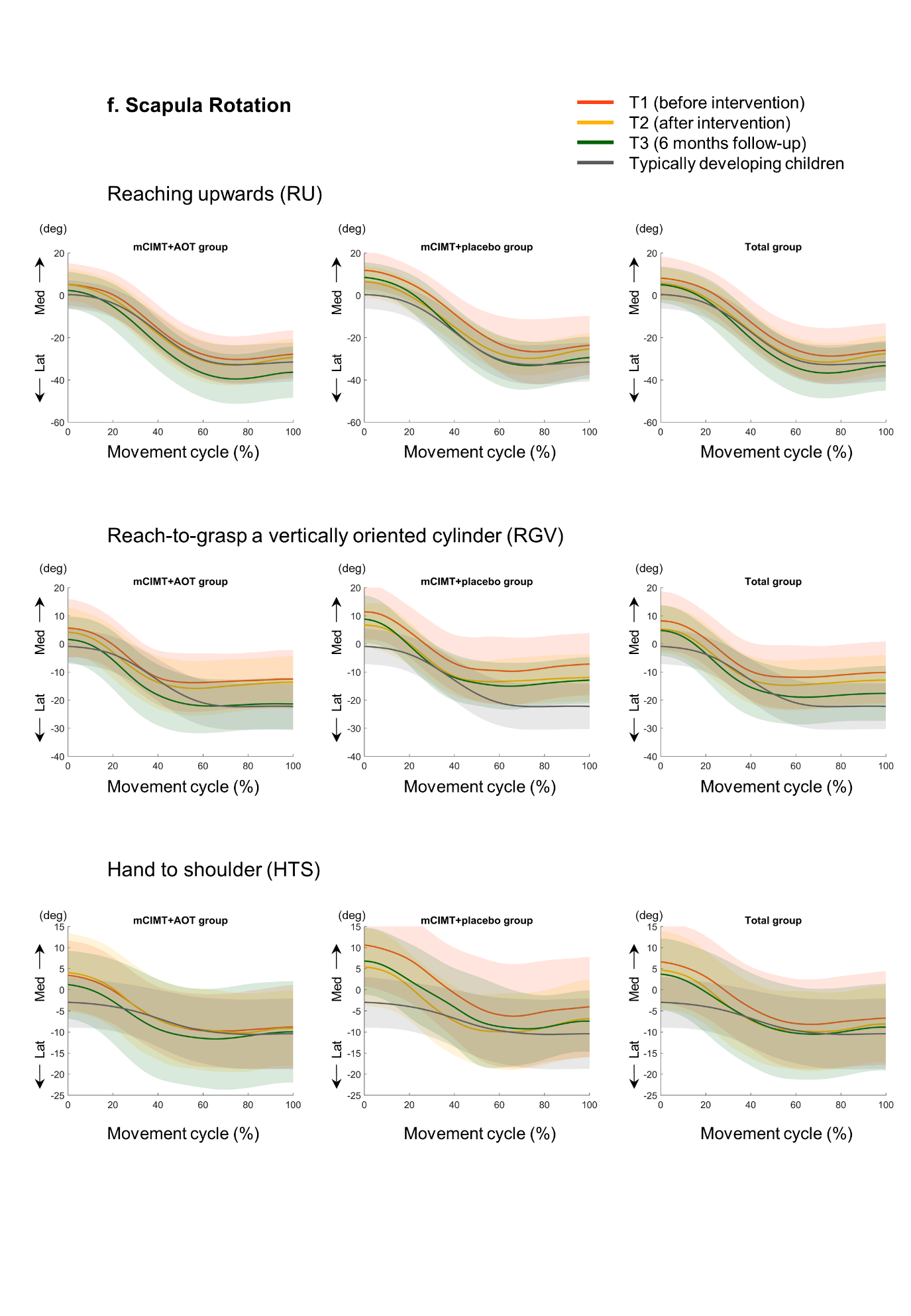


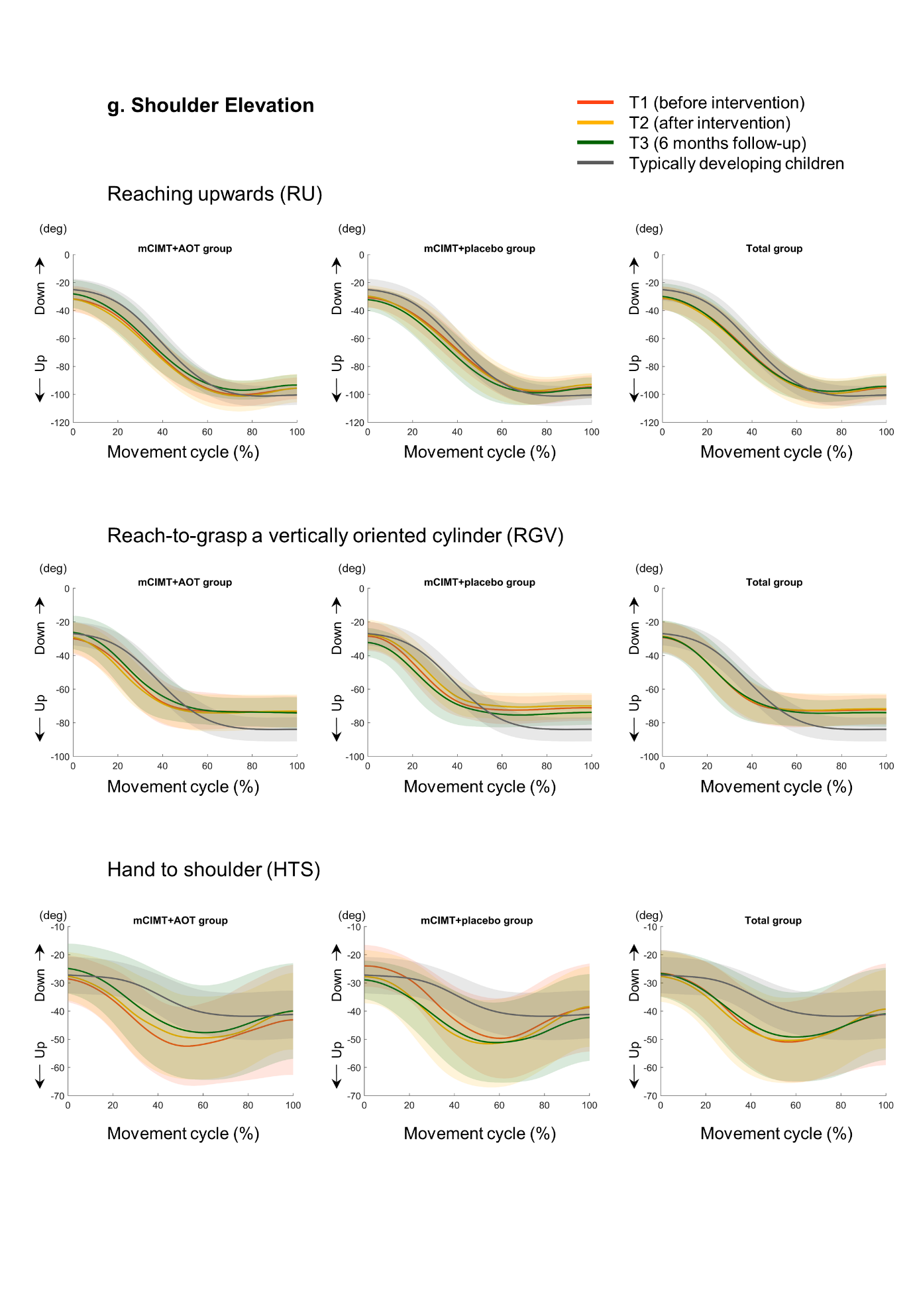


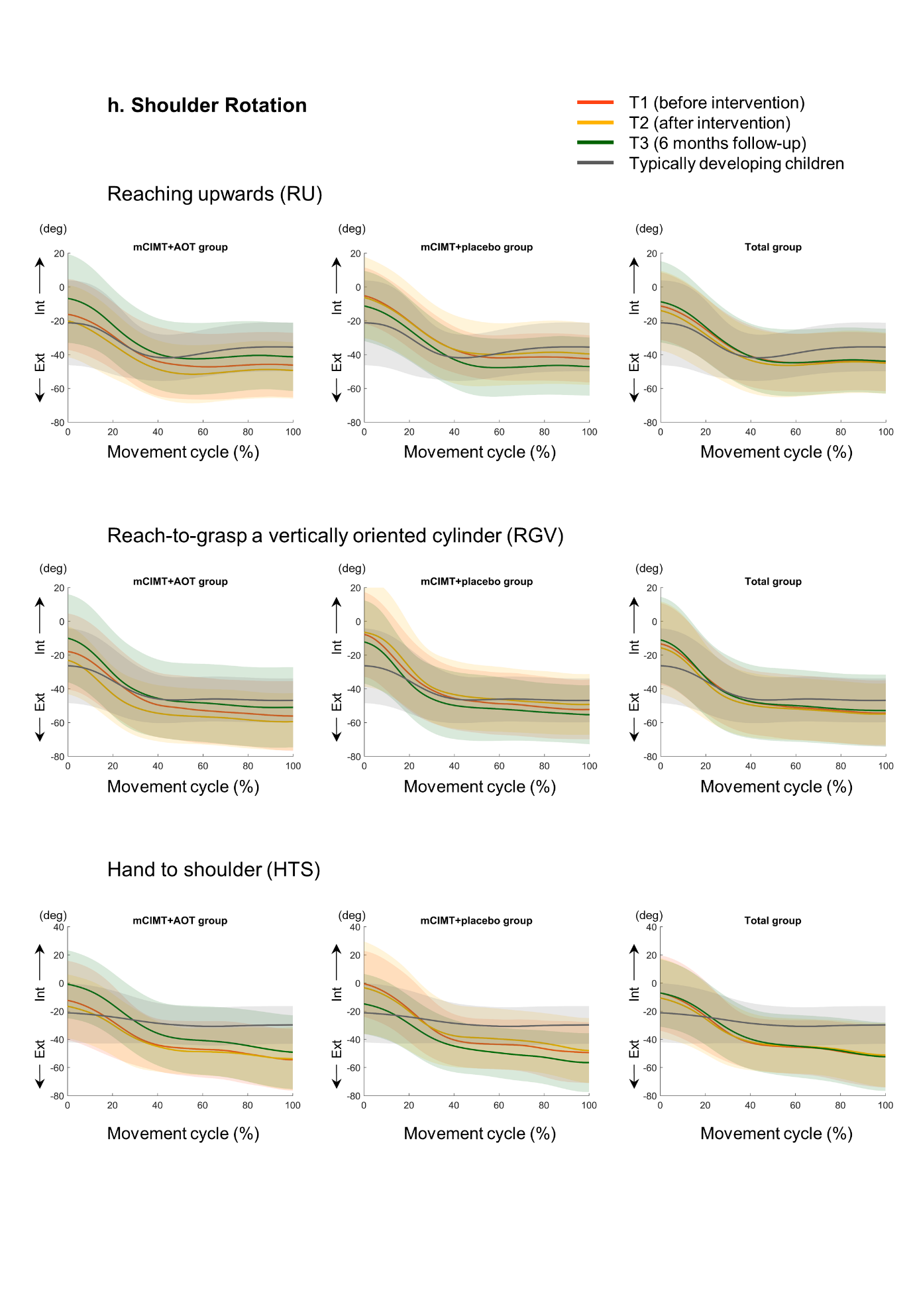


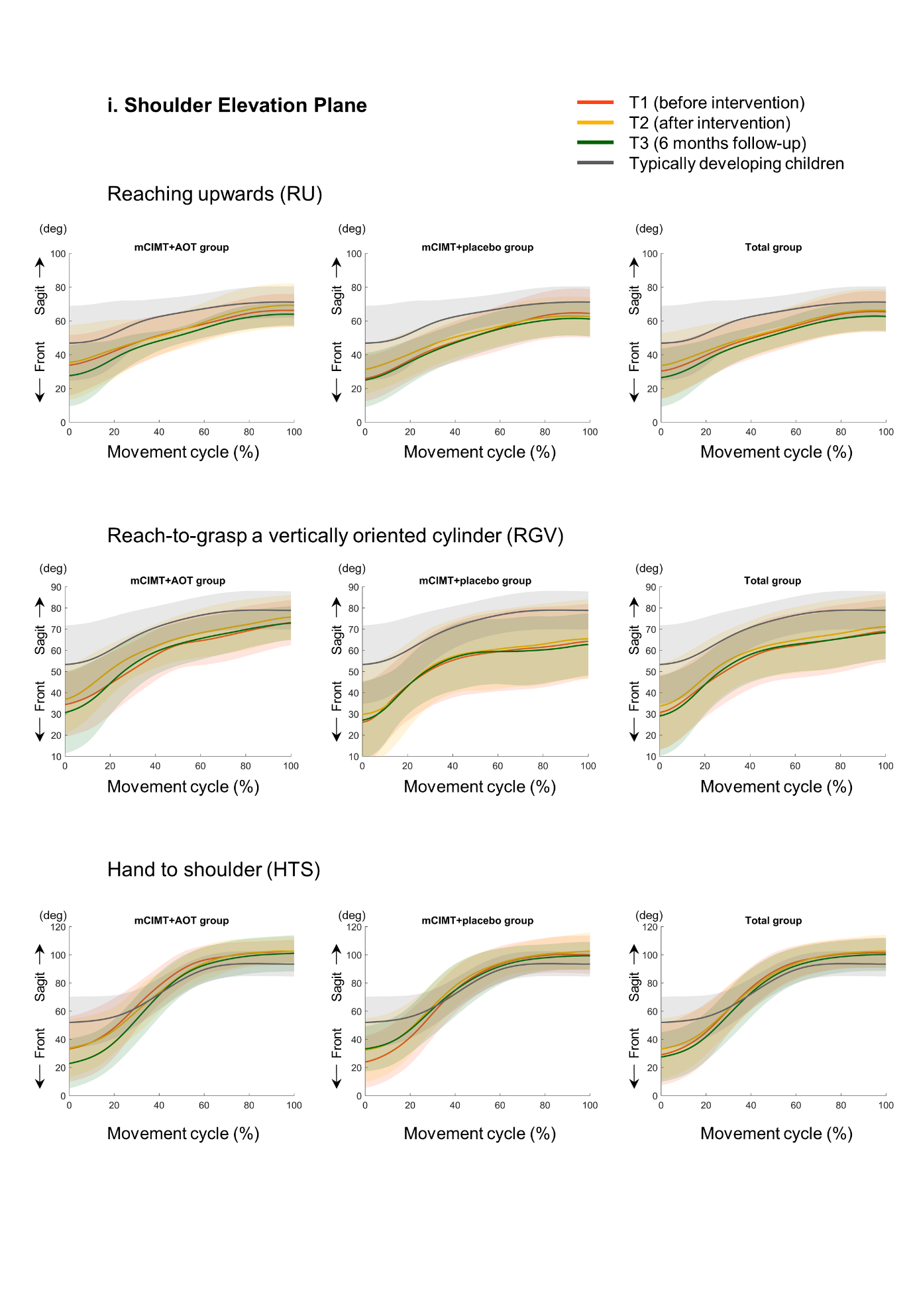


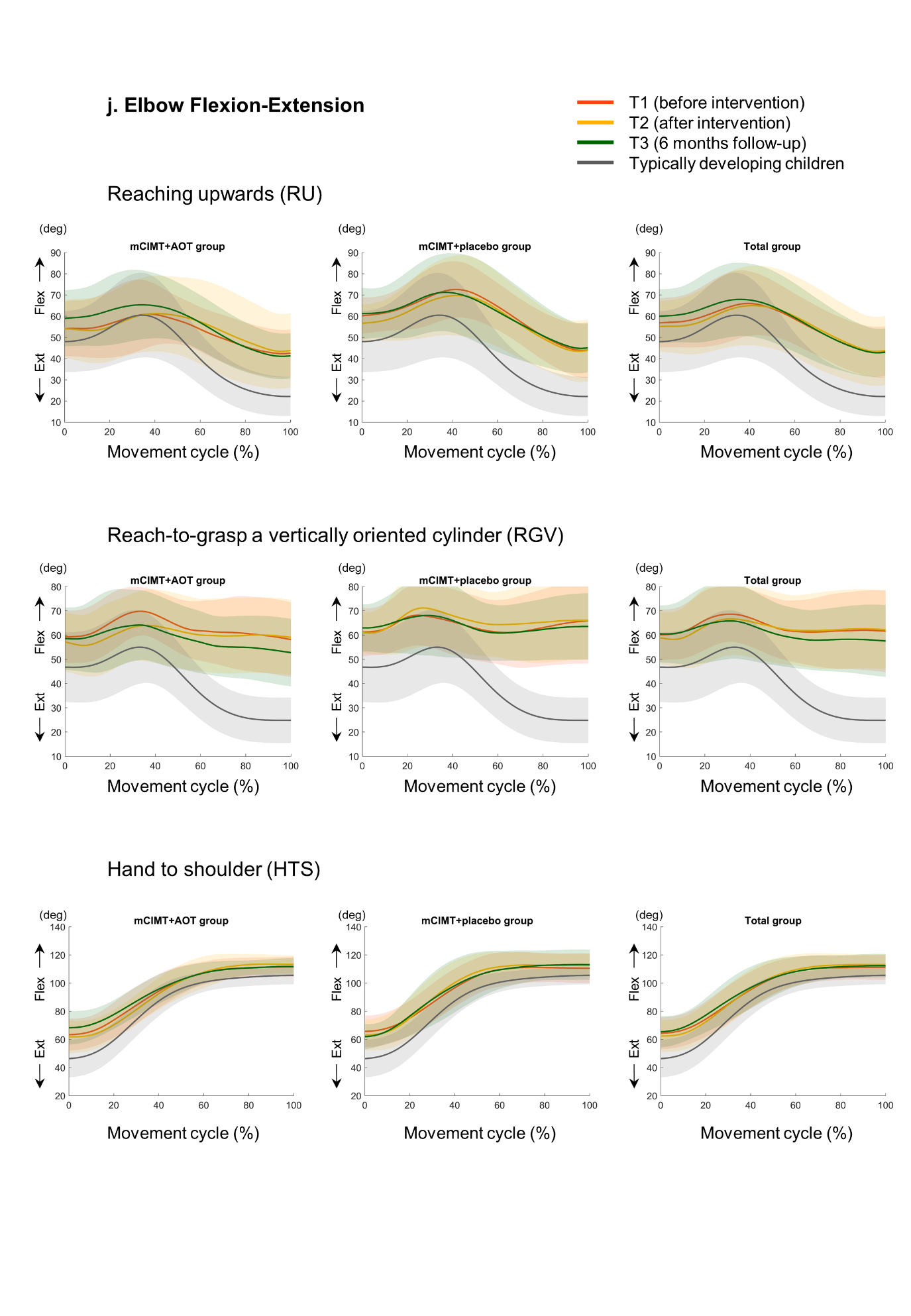


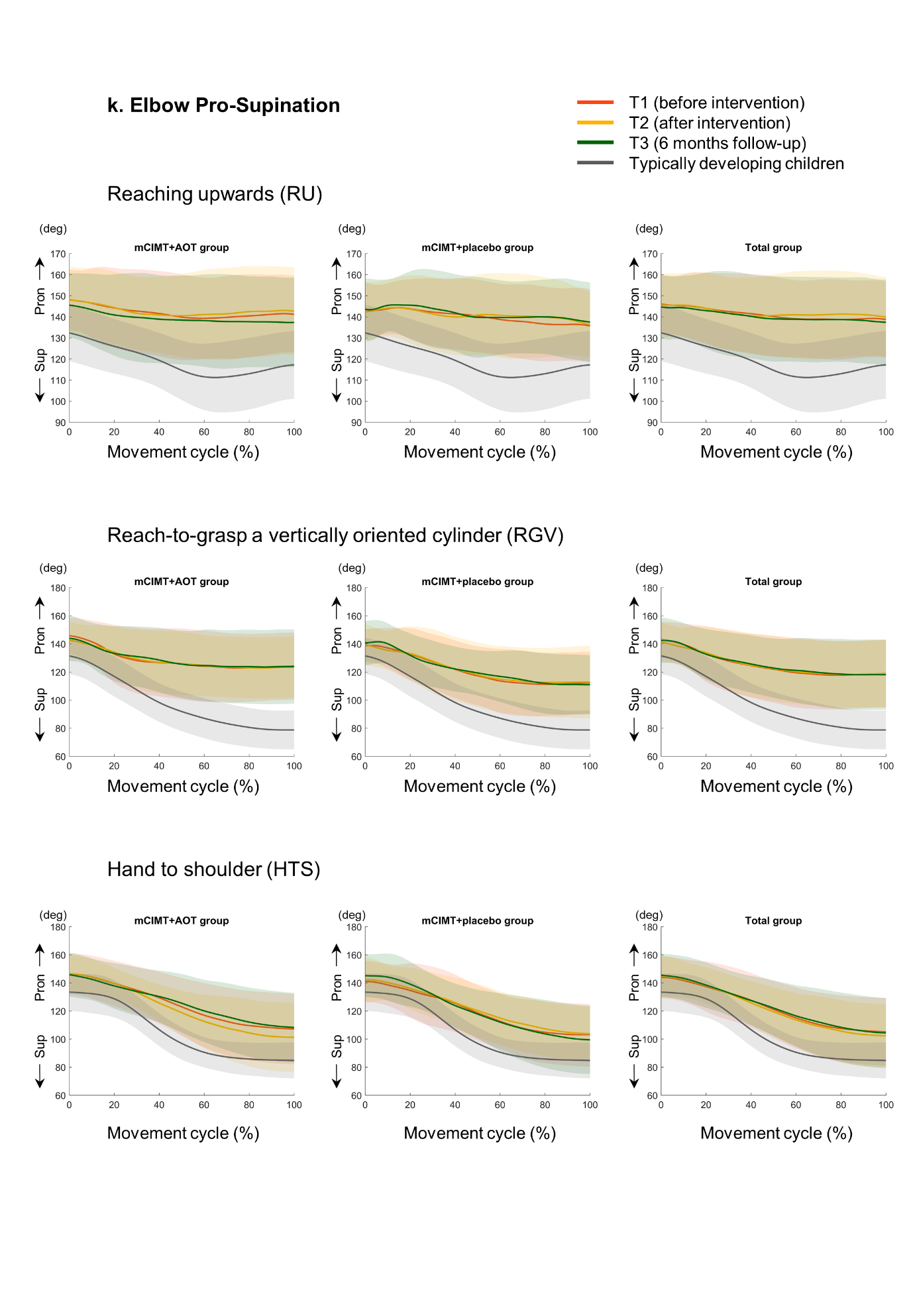


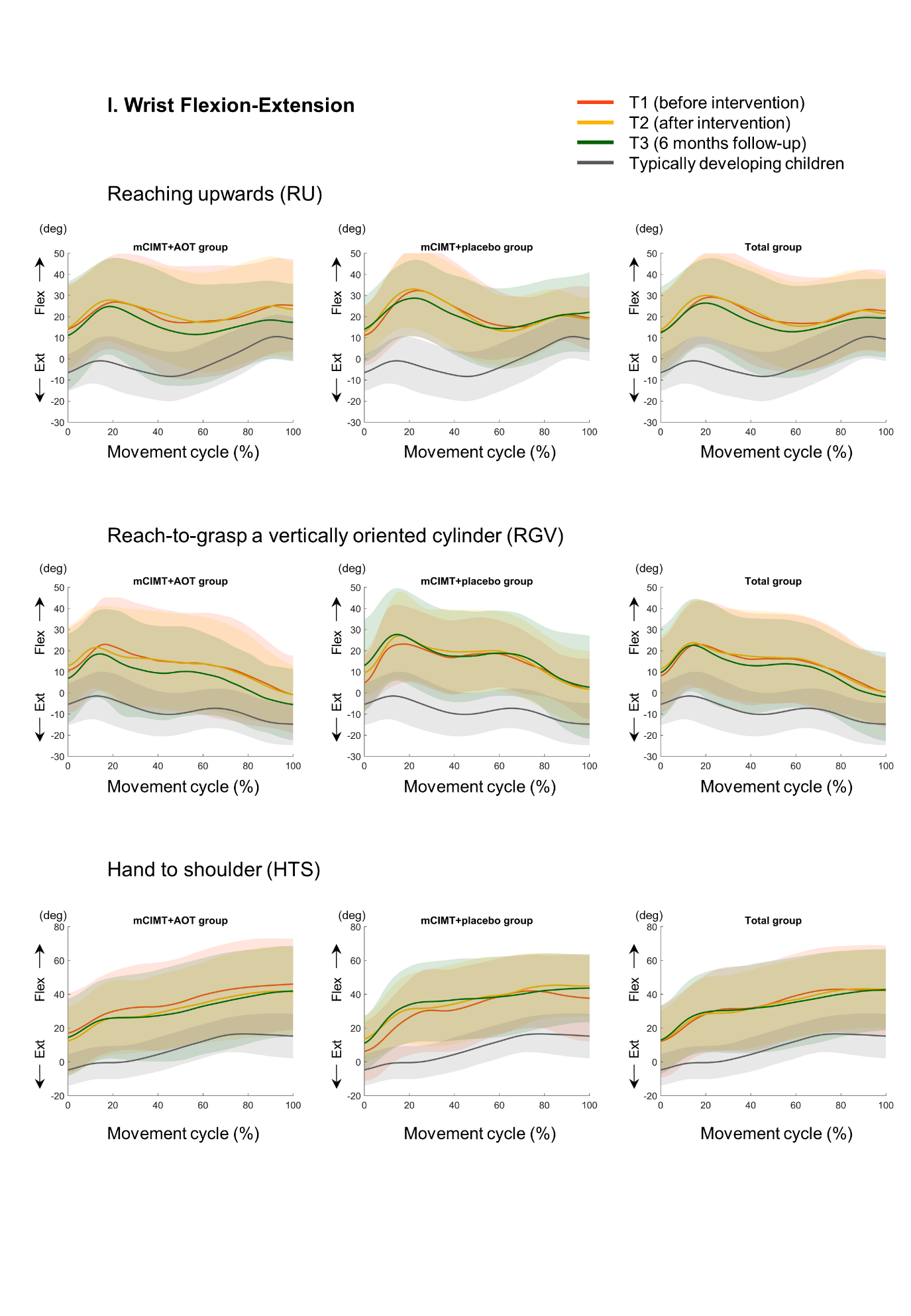


**Figure S3.** Correlation analyses between the change in kinematic and clinical measures at T1-T2 (left) and T1-T3 (right).


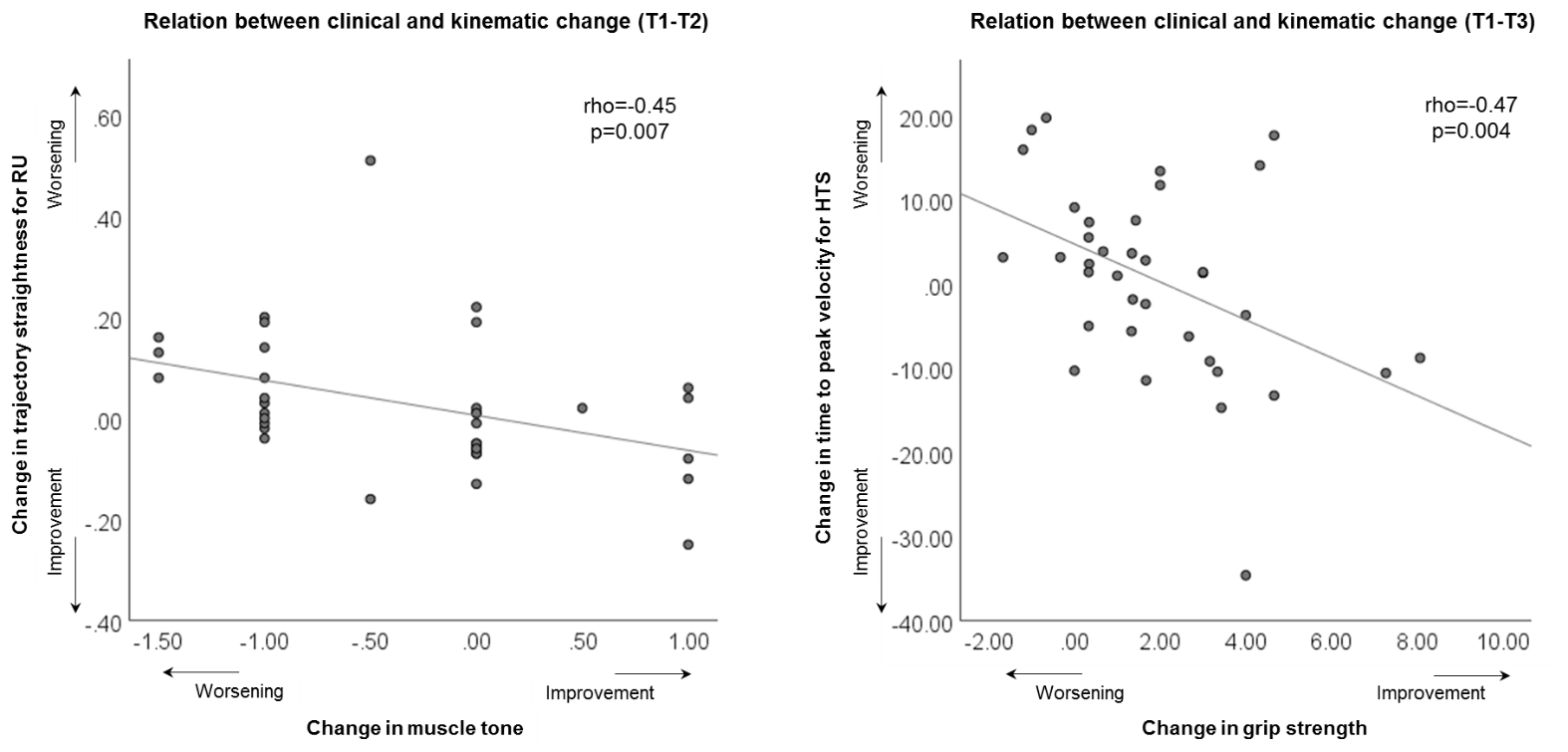

Supplement: Supplementary file 3 — Supplementary information 3. [file 41598_2020_67427_MOESM3_ESM.docx]
